# Supplementary material for: Microglial replacement in a Sandhoff disease mouse model reveals myeloid-derived β-hexosaminidase is necessary for neuronal health
Source: Nat Commun. 2025 Aug 27;16:7994. doi: 10.1038/s41467-025-63237-0 (PMC12391554; doi:10.1038/s41467-025-63237-0)
Supplement: Supplementary file 1 — Supplementary Information [file 41467_2025_63237_MOESM1_ESM.pdf]

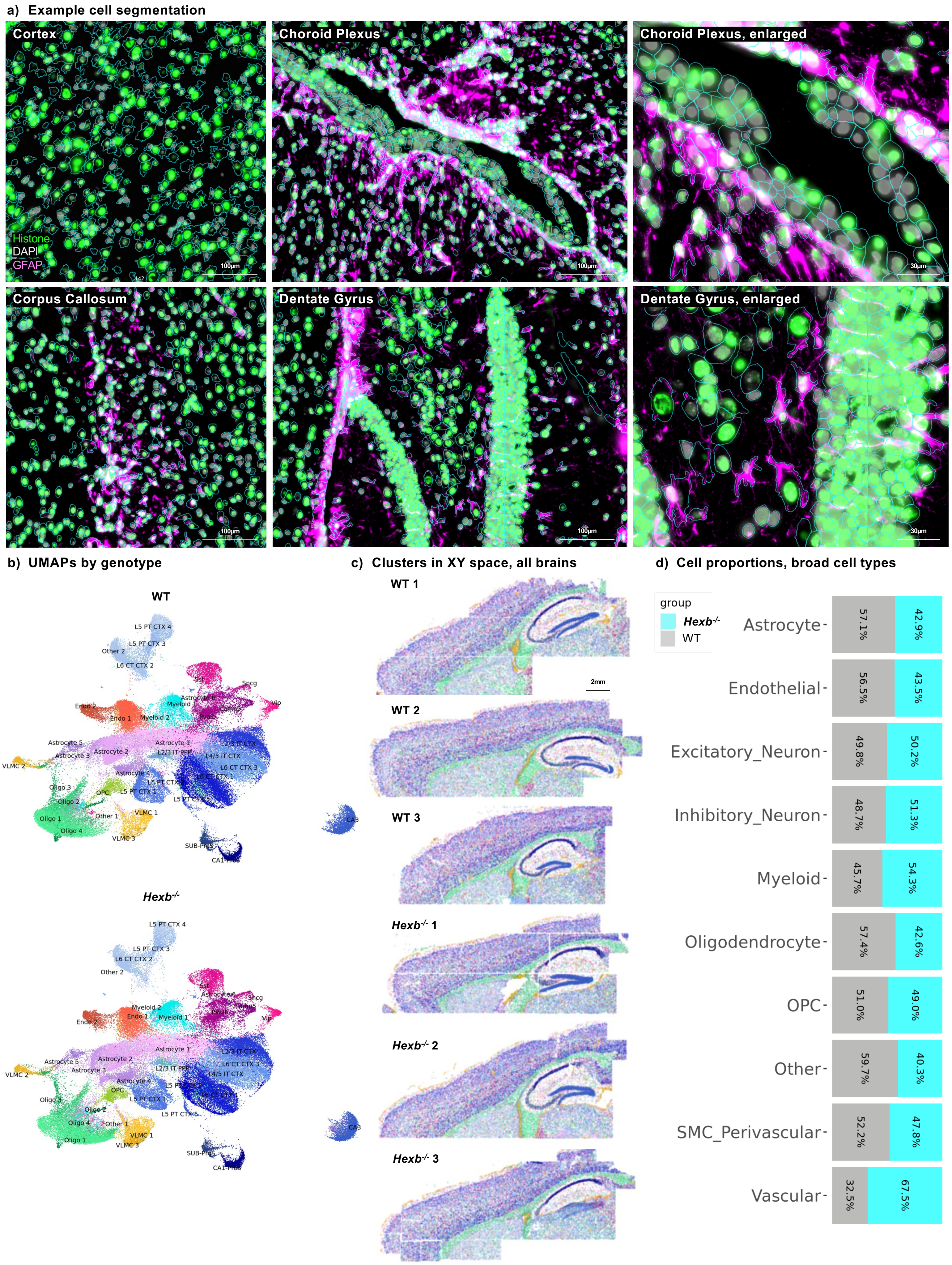

a) Example cell segmentation
Cortex
Choroid Plexus
Choroid Plexus, enlarged
Corpus Callosum
Dentate Gyrus
Dentate Gyrus, enlarged
Histone
DAPI
GFAP
100µm
30µm
b) UMAPs by genotype
WT
Hexb-/-
L5 PT CTX 4
L5 PT CTX 3
Other 2
L6 CT CTX 2
Sst
Sncg
Vip
Endo 2
Endo 1
Myeloid 1
Myeloid 2
Astrocyte 5
Astrocyte 3
Astrocyte 2
Astrocyte 1
Astrocyte 4
L2/3 IT CTX
L2/3 IT PPP
L4/5 IT CTX
L6 CT CTX 3
L5 PT CTX 1
L5 PT CTX 5
VLMC 2
VLMC 1
VLMC 3
Oligo 1
Oligo 2
Oligo 3
Oligo 4
OPC
Other 1
CA3
c) Clusters in XY space, all brains
WT 1
WT 2
WT 3
Hexb-/- 1
Hexb-/- 2
Hexb-/- 3
2mm
d) Cell proportions, broad cell types
group
Hexb-/-
WT
Astrocyte 57.1% 42.9%
Endothelial 56.5% 43.5%
Excitatory_Neuron 49.8% 50.2%
Inhibitory_Neuron 48.7% 51.3%
Myeloid 45.7% 54.3%
Oligodendrocyte 57.4% 42.6%
OPC 51.0% 49.0%
Other 59.7% 40.3%
SMC_Perivascular 52.2% 47.8%
Vascular 32.5% 67.5%

Supplemental Fig. 1: Spatial transcriptomic cell segmentation and expanded data visualization, *Hexb*$^{-/-}$ vs. WT.

(a) Representative images demonstrating cell segmentation in cortex, choroid plexus/ventricle, corpus callosum, and dentate gyrus. Cells were imaged with rRNA (not shown), Histone, DAPI, and GFAP markers and segmented automatically. (b) Uniform Manifold Approximation and Projection (UMAP) of 39 clusters split by group. (c) 39 clusters plotted in XY space in all 6 brains from WT control and *Hexb*$^{-/-}$ control brains (n=3/group). (d) Bar graph of proportions of cell counts by broad cell type per group.

a) **Top 5 marker genes per subcluster**

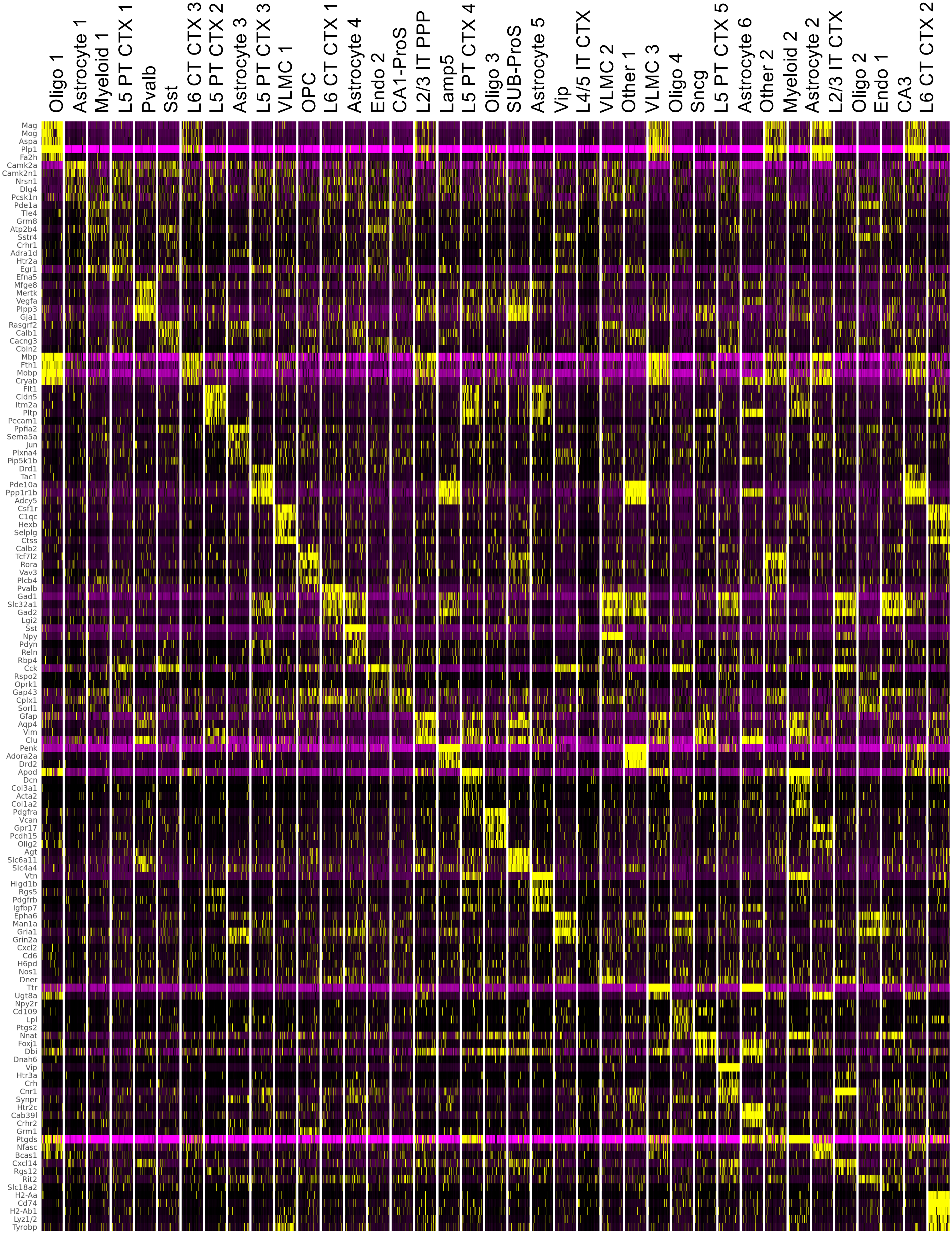

Supplemental Fig. 2: Heatmap of top 5 marker genes for all spatial transcriptomics subclusters, *Hexb*$^{-/-}$ vs. WT.

(a) Heatmap of top 5 marker genes for each subcluster.

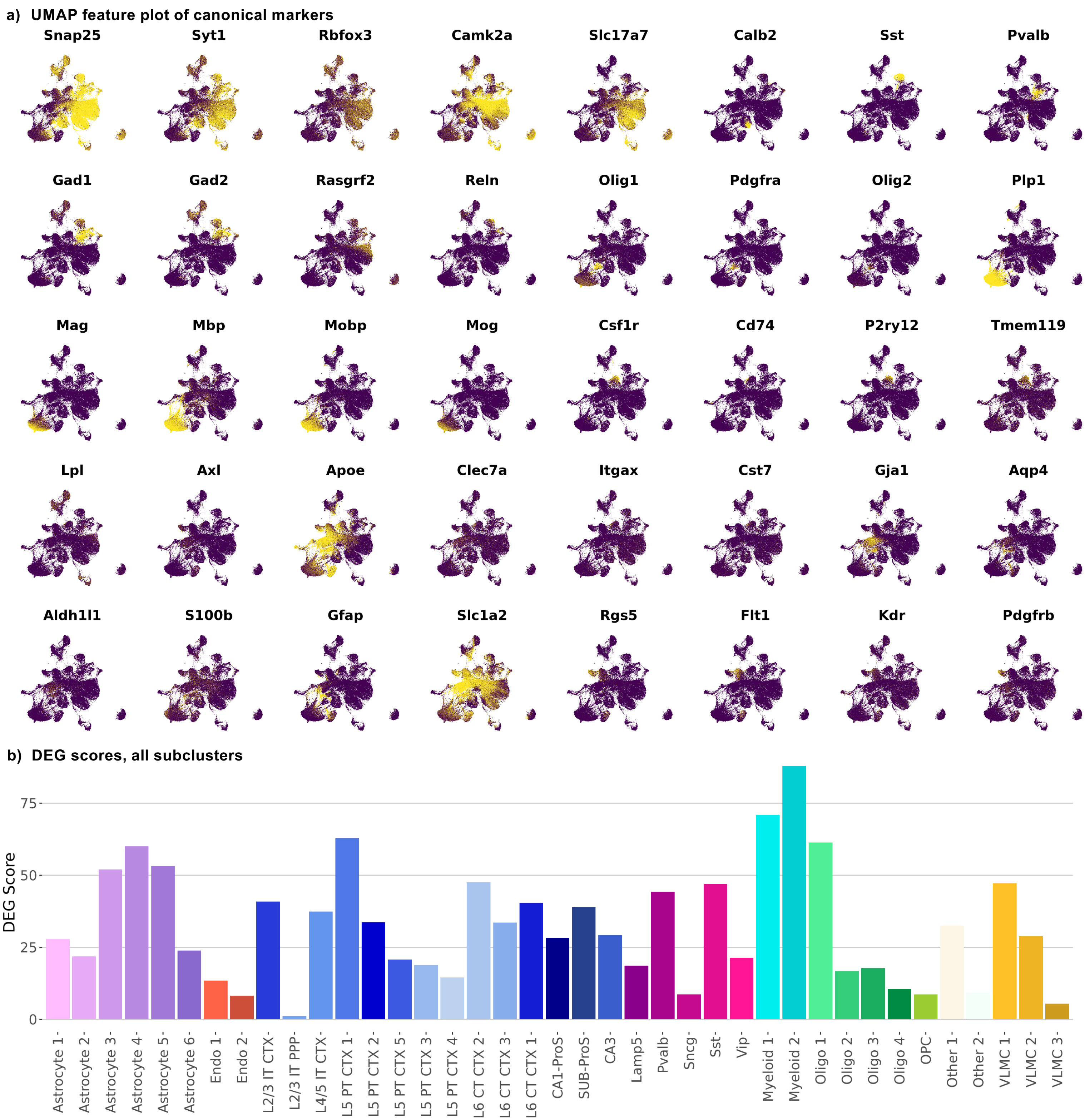
a) UMAP feature plot of canonical markers
Snap25
Syt1
Rbfox3
Camk2a
Slc17a7
Calb2
Sst
Pvalb
Gad1
Gad2
Rasgrf2
Reln
Olig1
Pdgfra
Olig2
Plp1
Mag
Mbp
Mobp
Mog
Csf1r
Cd74
P2ry12
Tmem119
Lpl
Axl
Apoe
Clec7a
Itgax
Cst7
Gja1
Aqp4
Aldh1l1
S100b
Gfap
Slc1a2
Rgs5
Flt1
Kdr
Pdgfrb
b) DEG scores, all subclusters
DEG Score
0
25
50
75
Astrocyte 1
Astrocyte 2
Astrocyte 3
Astrocyte 4
Astrocyte 5
Astrocyte 6
Endo 1
Endo 2
L2/3 IT CTX
L2/3 IT PPP
L4/5 IT CTX
L5 PT CTX 1
L5 PT CTX 2
L5 PT CTX 5
L5 PT CTX 3
L5 PT CTX 4
L6 CT CTX 2
L6 CT CTX 3
L6 CT CTX 1
CA1-ProS
SUB-ProS
CA3
Lamp5
Pvalb
Sncg
Sst
Vip
Myeloid 1
Myeloid 2
Oligo 1
Oligo 2
Oligo 3
Oligo 4
OPC
Other 1
Other 2
VLMC 1
VLMC 2
VLMC 3

Supplemental Fig. 3: Subcluster annotation information and DEG scores for all clusters identified by spatial transcriptomics, *Hexb*$^{-/-}$ vs. WT.

(a) Uniform Manifold Approximation and Projection (UMAP) of 39 clusters showing transcript expression of canonical marker genes for different broad cell types (purple = low, yellow = high). (b) Bar graph of differentially expressed gene (DEG) scores in all subclusters. Following differential gene expression analysis, DEG score was calculated using the DEGs from treatment condition pairs (i.e., BMT-treated *Hexb*$^{-/-}$ vs. WT control, BMT + CSF1Ri-treated *Hexb*$^{-/-}$ vs. WT control) in each subcluster by summing the absolute value of the $\log_2$ fold change values for all DEGs identified between WT control and BMT-treated *Hexb*$^{-/-}$ or BMT + CSF1Ri-treated *Hexb*$^{-/-}$ with a p_{adj} value below 0.05.

a) Volcano plots, *Hexb*$^{-/-}$ versus WT, all subclusters

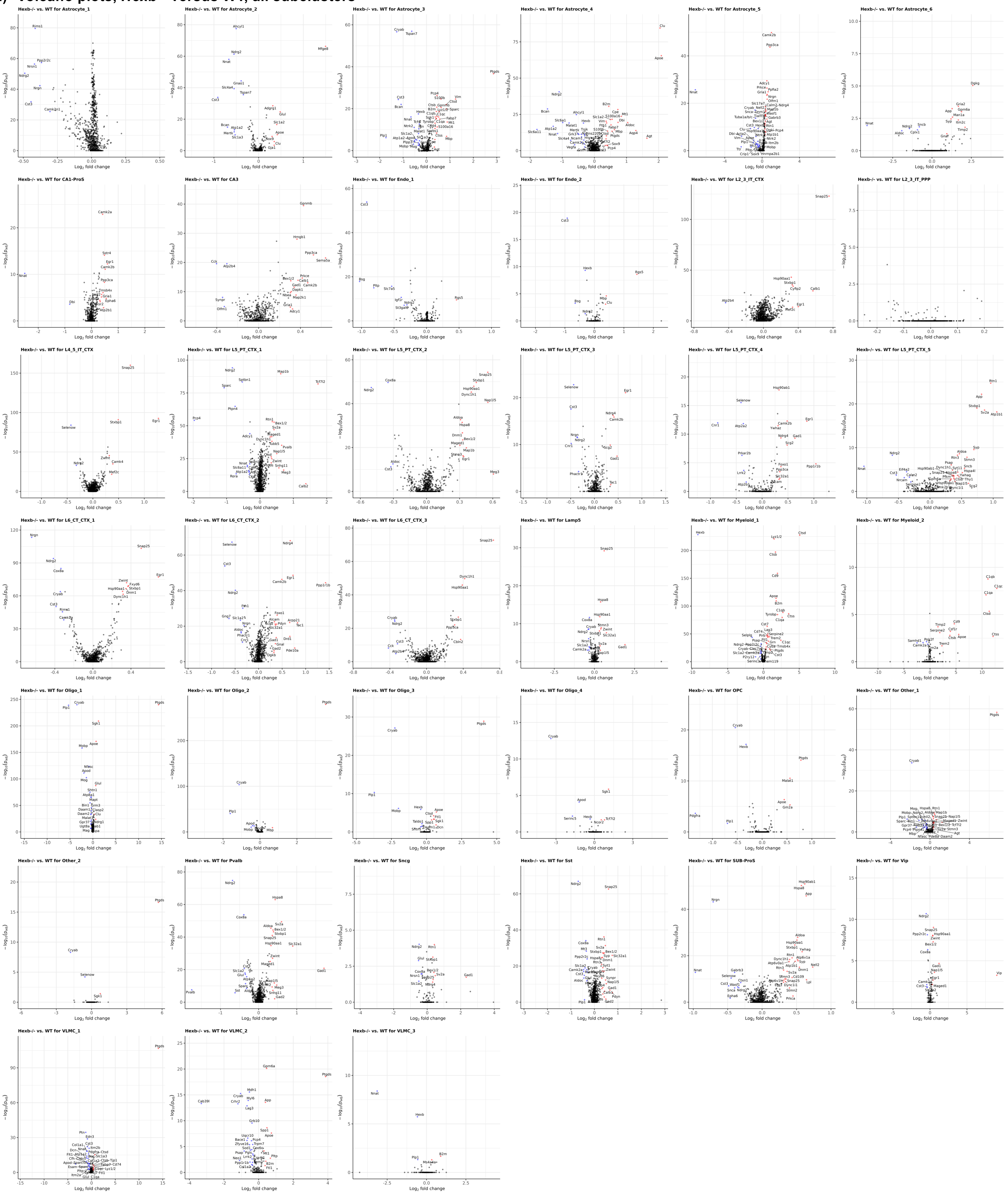

Supplemental Fig. 4: Volcano plots for all spatial transcriptomics subclusters, *Hexb*$^{-/-}$ vs. WT.

(a) Volcano plots of differentially expressed genes (DEGs) between *Hexb*$^{-/-}$ and WT control for all cellular subclusters.

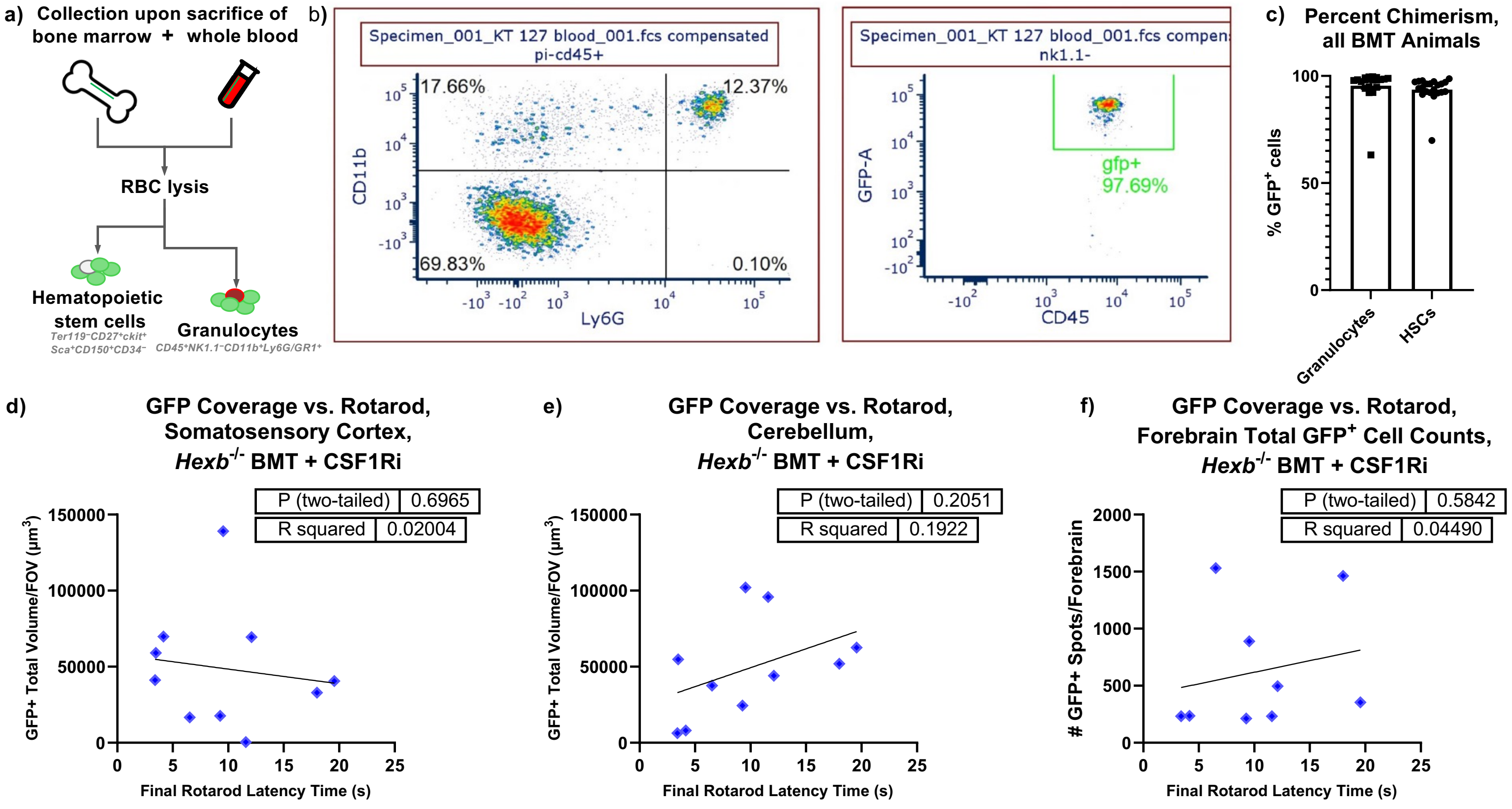
a) Collection upon sacrifice of bone marrow + whole blood
RBC lysis
Hematopoietic stem cells
Ter119⁻CD27⁺ckit⁺ Sca⁺CD150⁺CD34⁻
Granulocytes
CD45⁺NK1.1⁻CD11b⁺Ly6G/GR1⁺
b)
Specimen_001_KT 127 blood_001.fcs compensated
pi-cd45+
17.66%
12.37%
69.83%
0.10%
CD11b
Ly6G
Specimen_001_KT 127 blood_001.fcs compen
nk1.1-
gfp+
97.69%
GFP-A
CD45
c) Percent Chimerism, all BMT Animals
% GFP+ cells
Granulocytes
HSCs
d) GFP Coverage vs. Rotarod, Somatosensory Cortex, Hexb-/- BMT + CSF1Ri
P (two-tailed) 0.6965
R squared 0.02004
GFP+ Total Volume/FOV (µm³)
Final Rotarod Latency Time (s)
e) GFP Coverage vs. Rotarod, Cerebellum, Hexb-/- BMT + CSF1Ri
P (two-tailed) 0.2051
R squared 0.1922
GFP+ Total Volume/FOV (µm³)
Final Rotarod Latency Time (s)
f) GFP Coverage vs. Rotarod, Forebrain Total GFP+ Cell Counts, Hexb-/- BMT + CSF1Ri
P (two-tailed) 0.5842
R squared 0.04490
GFP+ Spots/Forebrain
Final Rotarod Latency Time (s)

Supplemental Fig. 5: Confirmation of successful BMT by flow cytometry and correlation analysis of regional peripheral cell infiltration versus final week Rotarod score.

(a) Schematic of sample collection for assessment of donor chimerism. Whole bone marrow and whole blood were collected from chimeric mice at point of sacrifice. Red blood cells (RBCs) were lysed to enrich for hematopoietic stem cells (HSC; $Ter119^{-}CD27^{+}ckit^{+}Sca^{+}CD150^{+}CD34^{-}$ cells) In bone marrow and granulocytes ($CD45^{+}NK1.1^{-}CD11b^{+}GR1/Ly6G^{+}$ cells) in blood. (b) Representative flow cytometry plot showing gating strategy for granulocytes. (c) Bar graph of percent donor chimerism of all BMT (BMT-treated WT, BMT + CSF1Ri-treated WT, *Hexb*$^{-/-}$, BMT-treated *Hexb*$^{-/-}$, and BMT + CSF1Ri-treated *Hexb*$^{-/-}$) mice. Donor chimerism was assessed by % green fluorescent protein (GFP)$^{+}$ cells (GFP^{+} cells/total cells). (d-e) Scatterplots with line of best fit of final week (week 16) Rotarod latency-to-fall time (x axis) versus total GFP^{+} staining volume (y axis) in 20x confocal FOVs from (d) somatosensory cortex and (e) cerebellum in BMT + CSF1Ri-treated *Hexb*$^{-/-}$ mice. (f) Scatterplot with line of best fit of final week (week 16) Rotarod latency-to-fall time (x axis) versus number of green fluorescent (GFP)$^{+}$ spots/cells (y axis) in forebrain portion of whole brain sagittal scans of BMT + CSF1Ri-treated *Hexb*$^{-/-}$ mice. n=9 WT BMT; n=10 WT BMT, *Hexb*$^{-/-}$, *Hexb*$^{-/-}$ BMT + CSF1Ri; n=11 WT, *Hexb*$^{-/-}$ BMT.

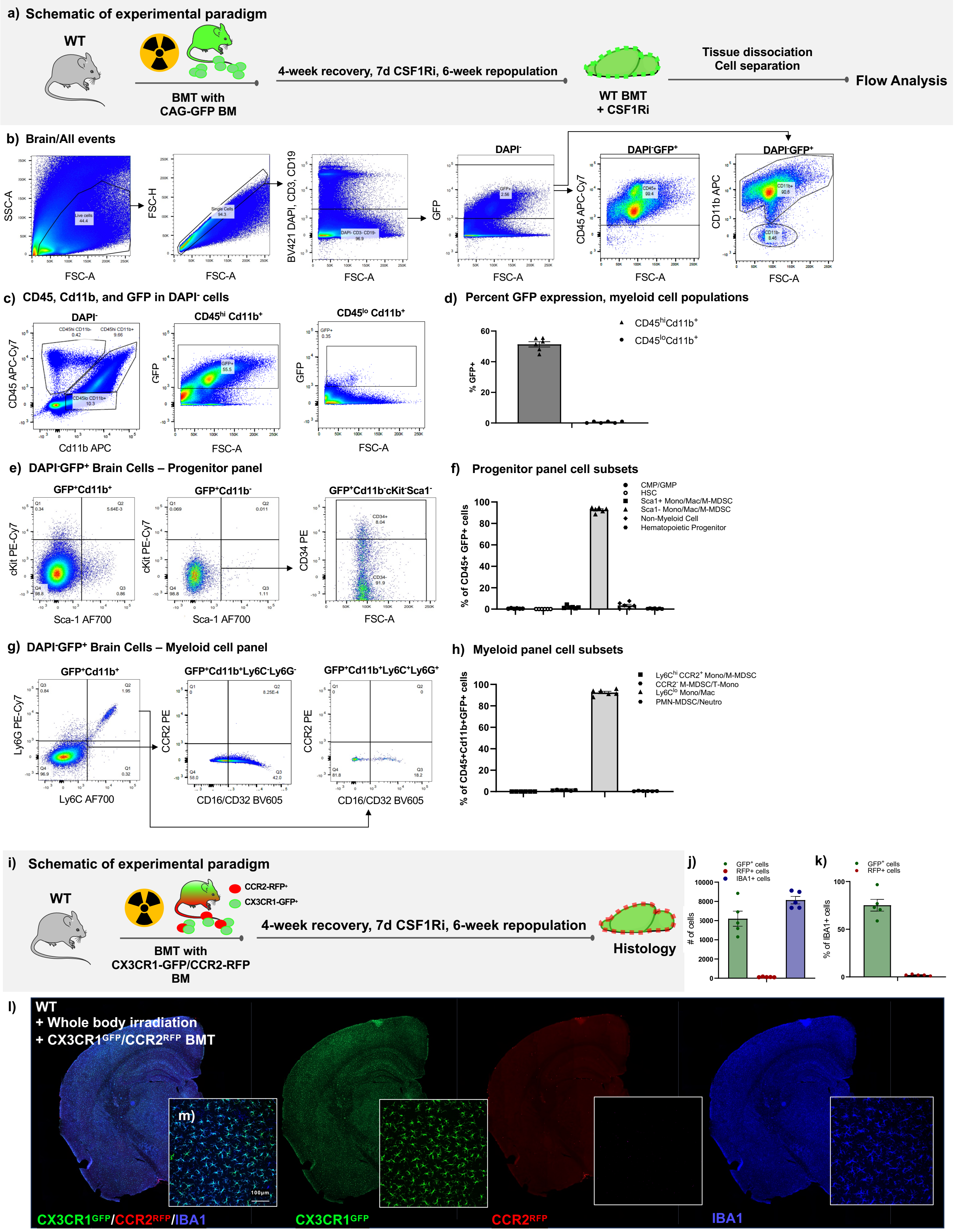

a) Schematic of experimental paradigm
WT
BMT with CAG-GFP BM
4-week recovery, 7d CSF1Ri, 6-week repopulation
WT BMT + CSF1Ri
Tissue dissociation Cell separation
Flow Analysis
b) Brain/All events
SSC-A
FSC-A
Live cells 44.4
FSC-H
Single Cells 94.3
BV421 DAPI, CD3, CD19
DAPI- CD3- CD19- 96.9
DAPI-
GFP
GFP+ 2.56
DAPI-GFP+
CD45 APC-Cy7
CD45+ 99.4
CD11b APC
CD11b+ 90.8
CD11b- 8.46
c) CD45, Cd11b, and GFP in DAPI- cells
CD45hi CD11b- 0.42
CD45hi CD11b+ 9.66
CD45lo CD11b+ 10.3
Cd11b APC
CD45hi Cd11b+
GFP+ 55.5
CD45lo Cd11b+
GFP+ 0.35
d) Percent GFP expression, myeloid cell populations
CD45hiCd11b+
CD45loCd11b+
% GFP+
e) DAPI-GFP+ Brain Cells – Progenitor panel
GFP+Cd11b+
GFP+Cd11b-
GFP+Cd11b-cKit-Sca1-
cKit PE-Cy7
Sca-1 AF700
CD34 PE
CD34+ 8.04
CD34- 91.9
f) Progenitor panel cell subsets
CMP/GMP
HSC
Sca1+ Mono/Mac/M-MDSC
Sca1- Mono/Mac/M-MDSC
Non-Myeloid Cell
Hematopoietic Progenitor
% of CD45+ GFP+ cells
g) DAPI-GFP+ Brain Cells – Myeloid cell panel
GFP+Cd11b+
GFP+Cd11b+Ly6C-Ly6G-
GFP+Cd11b+Ly6C+Ly6G+
Ly6G PE-Cy7
Ly6C AF700
CCR2 PE
CD16/CD32 BV605
h) Myeloid panel cell subsets
Ly6Chi CCR2+ Mono/M-MDSC
CCR2- M-MDSC/T-Mono
Ly6Clo Mono/Mac
PMN-MDSC/Neutro
% of CD45+Cd11b+GFP+ cells
i) Schematic of experimental paradigm
WT
CCR2-RFP+
CX3CR1-GFP+
BMT with CX3CR1-GFP/CCR2-RFP BM
4-week recovery, 7d CSF1Ri, 6-week repopulation
Histology
j)
GFP+ cells
RFP+ cells
IBA1+ cells
# of cells
k)
% of IBA1+ cells
l)
WT
+ Whole body irradiation
+ CX3CR1GFP/CCR2RFP BMT
m)
100μm
CX3CR1GFP/CCR2RFP/IBA1
CX3CR1GFP
CCR2RFP
IBA1

Supplemental Fig. 6: Identification of infiltrating bone marrow-derived population by flow cytometry and immunohistochemistry. (a) Schematic of treatment paradigm. 12 mice were used, and 2 brains were pooled to comprise one n; total n=6. (b) Representative pseudocolor plots showing an overview of the common gating strategy. Cells were split between two panels which each used the same initial gating strategy. (c) Representative pseudocolor plots showing all DAPI- cells gated on CD45 and Cd11b, then gated on GFP for both CD45^{hi}Cd11b^{+} and CD45^{lo}Cd11b^{+} populations. (d) Bar graph showing the percentage of CD45^{hi}Cd11b^{+} and CD45^{lo}Cd11b^{+} cells that were GFP^{+}. (e) Representative pseudocolor plots for panel 1 (progenitor identification panel, CD45, CD11b, Sca-1, cKit, CD34) showing gating for cKit and Sca-1 in all GFP^{+}CD45^{hi} cells in both Cd11b^{+} and Cd11b^{-} populations. Cd11b^{-}Sca1^{-}cKit^{-} cells were then gated on CD34. (f) Bar graph showing the percentage of CD45^{hi}GFP^{+} cells identified as common myeloid progenitors/granulocyte monocyte progenitors (CMP/GMP; CD45^{hi}Cd11b^{+}cKit^{+}Sca-1^{-}), hematopoietic stem cells (HSC; CD45^{hi}Cd11b^{+}cKit^{+}Sca-1^{+}), Sca-1^{+} monocyte, macrophage, or monocytic myeloid-derived suppressor cells (Sca-1^{+} mono/mac/M-MDSC; CD45^{hi}Cd11b^{+}cKit^{-} Sca-1^{+}), Sca-1^{-} monocyte, macrophage, or monocytic myeloid-derived suppressor cells (Sca-1^{-} mono/mac/M-MDSC; CD45^{hi}Cd11b^{+}cKit^{-}Sca-1^{-}), non-myeloid cells (CD45^{hi}Cd11b^{-}cKit^{-}Sca-1^{-} CD34^{-}), or hematopoietic precursors (CD45^{hi}Cd11b^{-}cKit^{-}Sca-1^{-}CD34^{+}). (g) Representative pseudocolor plots for panel 2 (myeloid cell identification panel, CD45, Cd11b, Ly6C, Ly6G, CCR2, CD16/32) showing gating for Ly6C and Ly6G in all GFP^{+}CD45^{hi}Cd11b^{+} cells. Ly6C^{-}Ly6G^{-}, Ly6C^{+}Ly6G^{-}, and Ly6C^{+}Ly6G^{+} cells were then gated on CCR2 and CD16/32. (h) Bar graph showing the percentage of ^{i}GFP^{+}CD45^{h}Cd11b^{+} cells identified as Ly6C^{hi} monocytes or M-MDSCs (Ly6C^{hi} Mono/M-MDSC; CD45^{hi}Cd11b^{+}Ly6C^{hi}Ly6G^{-}CCR2^{+}), CCR2^{-} M-MDSCs or transitional monocytes (CCR2^{-} M-MDSC/T-Mono; CD45^{hi}Cd11b^{+}Ly6C^{hi}Ly6G^{-}CCR2^{-}), Ly6C^{lo} monocytes or macrophages (Ly6C^{lo} mono/mac; CD45^{hi}Cd11b^{+}Ly6C^{lo}Ly6G^{-}CCR2^{-}), or polymorphonuclear myeloid-derived suppressor cells or neutrophils (PMN-MDSC/Neutro; CD45^{hi}Cd11b^{+}Ly6C^{+}Ly6G^{+}CCR2^{-}). (i) Schematic of treatment paradigm. (j) Bar graph showing

quantification of total GFP+, RFP+, and IBA1+ (a myeloid cell identification marker) cell counts in whole brain coronal sections from WT BMT + CSF1Ri mice. (k) Bar graph showing quantification of percentage of IBA1+ cells expressing GFP or RFP. (l) Representative whole brain coronal sections and (m) 20x confocal images of the cortex stained for CX3CR1-GFP (green), CCR2-RFP (red), and IBA1 (blue) in WT BMT + CSF1Ri-treated *Hexb*$^{-/-}$ mice. Mouse images adapted from Servier Medical Art, https://smart.servier.com/smart_image/mouse/.

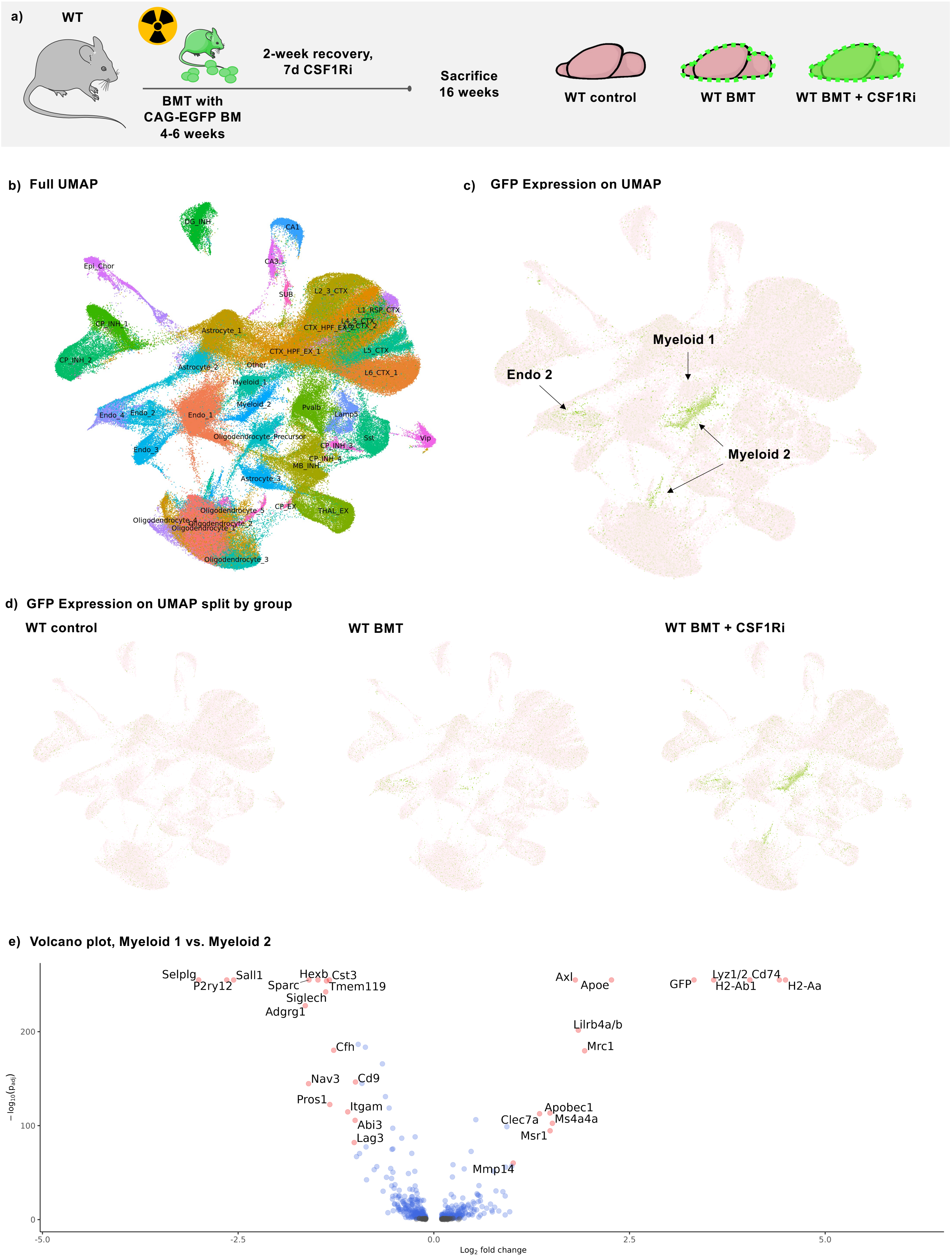
a)
WT
BMT with
CAG-EGFP BM
4-6 weeks
2-week recovery,
7d CSF1Ri
Sacrifice
16 weeks
WT control
WT BMT
WT BMT + CSF1Ri
b) Full UMAP
DG_INH
CA1
CA3
Epi_Chor
SUB
L2_3_CTX
L1_RSP_CTX
L4_5_CTX
CP_INH_1
Astrocyte_1
CTX_HPF_EX_2
L6_CTX_2
CTX_HPF_EX_1
L5_CTX
CP_INH_2
Astrocyte_2
Other
L6_CTX_1
Myeloid_1
Myeloid_2
Endo_4
Endo_2
Endo_1
Pvalb
Lamp5
Oligodendrocyte_Precursor
Sst
Vip
Endo_3
CP_INH_3
CP_INH_4
MB_INH
Astrocyte_3
Oligodendrocyte_5
CP_EX
THAL_EX
Oligodendrocyte_4
Oligodendrocyte_2
Oligodendrocyte_1
Oligodendrocyte_3
c) GFP Expression on UMAP
Myeloid 1
Endo 2
Myeloid 2
d) GFP Expression on UMAP split by group
WT control
WT BMT
WT BMT + CSF1Ri
e) Volcano plot, Myeloid 1 vs. Myeloid 2
Selplg
P2ry12
Sall1
Hexb
Cst3
Sparc
Tmem119
Siglech
Adgrg1
Axl
Apoe
GFP
Lyz1/2
Cd74
H2-Ab1
H2-Aa
Lilrb4a/b
Mrc1
Cfh
Nav3
Cd9
Pros1
Itgam
Abi3
Lag3
Apobec1
Ms4a4a
Clec7a
Msr1
Mmp14
200
100
0
$-\log_{10}(p_{adj})$
-5.0
-2.5
0.0
2.5
5.0
Log_2 fold change

Supplemental Fig. 7: Spatial transcriptomic analysis of engrafted GFP+ cells. (a) Schematic of treatment paradigm. WT mice (n=6) were split evenly into 3 groups: untreated control, bone marrow transplant (BMT), and bone marrow transplant plus colony stimulating factor 1 inhibitor treatment (BMT + CSF1Ri). Mice were sacrificed at 16 weeks. (b) Uniform Manifold Approximation and Projection (UMAP) of 279613 cells across 6 brains. Clustering at 1.0 resolution yielded 41 clusters, which were annotated manually based on gene expression and anatomical location in space. (c) Feature plot of GFP expression projected onto full UMAP. GFP-expressing cell types labeled with arrows. (d) Feature plots of GFP expression projected onto UMAPs for each group demonstrating that the majority of GFP cells are in the BMT + CSF1Ri group. (e) Volcano plot of differentially expressed genes (DEGs) between Myeloid 1 (GFP-) and Myeloid 2 (GFP+) cell types. Left: highly expressed genes in Myeloid 1; Right: highly expressed genes in Myeloid 2. Mouse images adapted from Servier Medical Art, https://smart.servier.com/smart_image/mouse/.

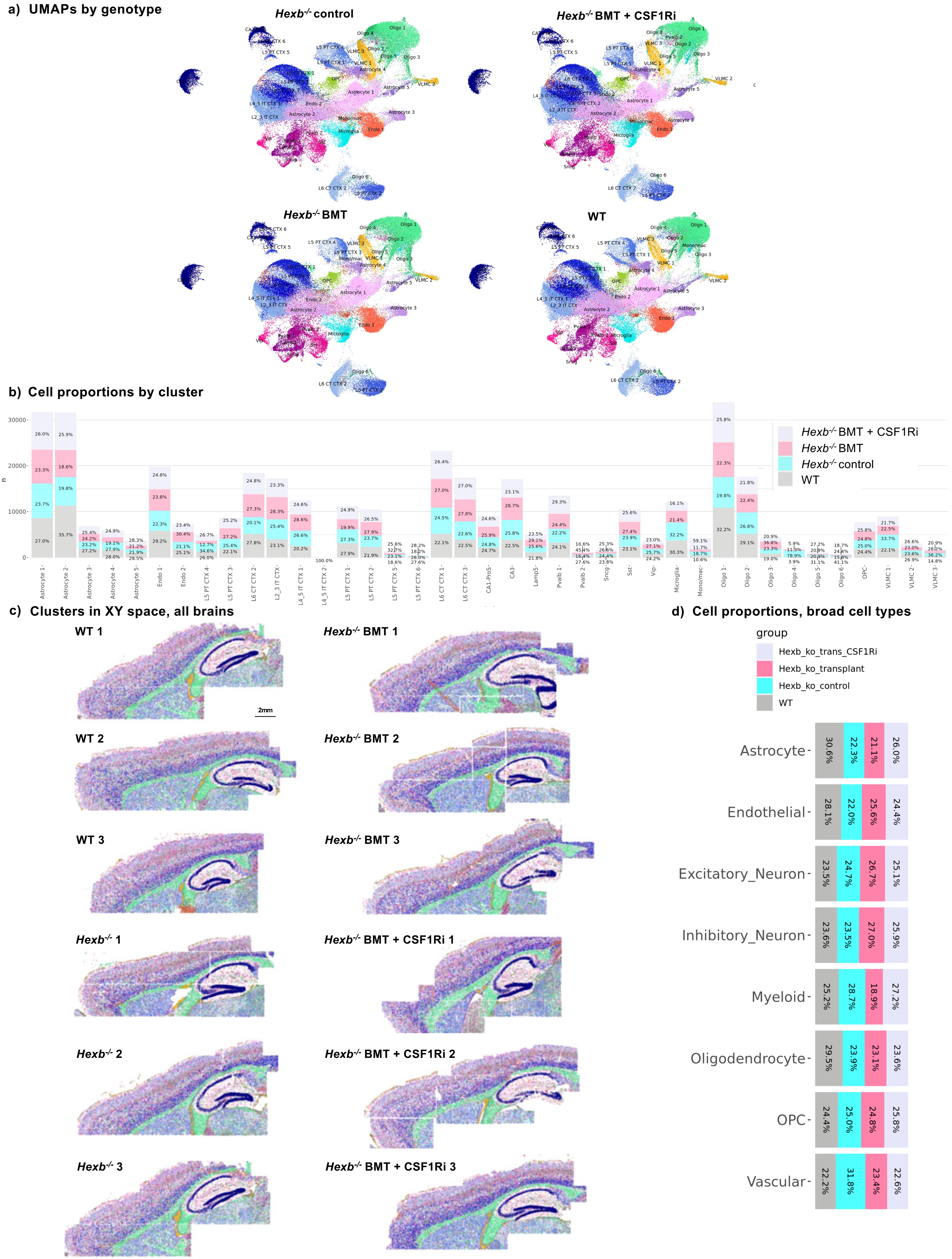
a) UMAPs by genotype
Hexb-/- control
Hexb-/- BMT + CSF1Ri
Hexb-/- BMT
WT
b) Cell proportions by cluster
Hexb-/- BMT + CSF1Ri
Hexb-/- BMT
Hexb-/- control
WT
c) Clusters in XY space, all brains
WT 1
WT 2
WT 3
Hexb-/- 1
Hexb-/- 2
Hexb-/- 3
Hexb-/- BMT 1
Hexb-/- BMT 2
Hexb-/- BMT 3
Hexb-/- BMT + CSF1Ri 1
Hexb-/- BMT + CSF1Ri 2
Hexb-/- BMT + CSF1Ri 3
2mm
d) Cell proportions, broad cell types
group
Hexb_ko_trans_CSF1Ri
Hexb_ko_transplant
Hexb_ko_control
WT
Astrocyte 30.6% 22.3% 21.1% 26.0%
Endothelial 28.1% 22.0% 25.6% 24.4%
Excitatory_Neuron 23.5% 24.7% 26.7% 25.1%
Inhibitory_Neuron 23.6% 23.5% 27.0% 25.9%
Myeloid 25.2% 28.7% 18.9% 27.2%
Oligodendrocyte 29.5% 23.9% 23.1% 23.6%
OPC 24.4% 25.0% 24.8% 25.8%
Vascular 22.2% 31.8% 23.4% 22.6%

Supplemental Fig. 8: Expanded spatial transcriptomic data visualization, WT and all *Hexb*$^{-/-}$ groups.

(a) Uniform Manifold Approximation and Projection (UMAP) of 38 clusters split by genotype and treatment condition group. (b) Bar graph of proportions of cell counts by broad cell type per group. (c) 38 clusters plotted in XY space in all 12 brains from WT control, *Hexb*$^{-/-}$ control, BMT-treated *Hexb*$^{-/-}$, and BMT + CSF1Ri-treated *Hexb*$^{-/-}$ brains (n=3/group).

a) Top 5 marker genes per subcluster

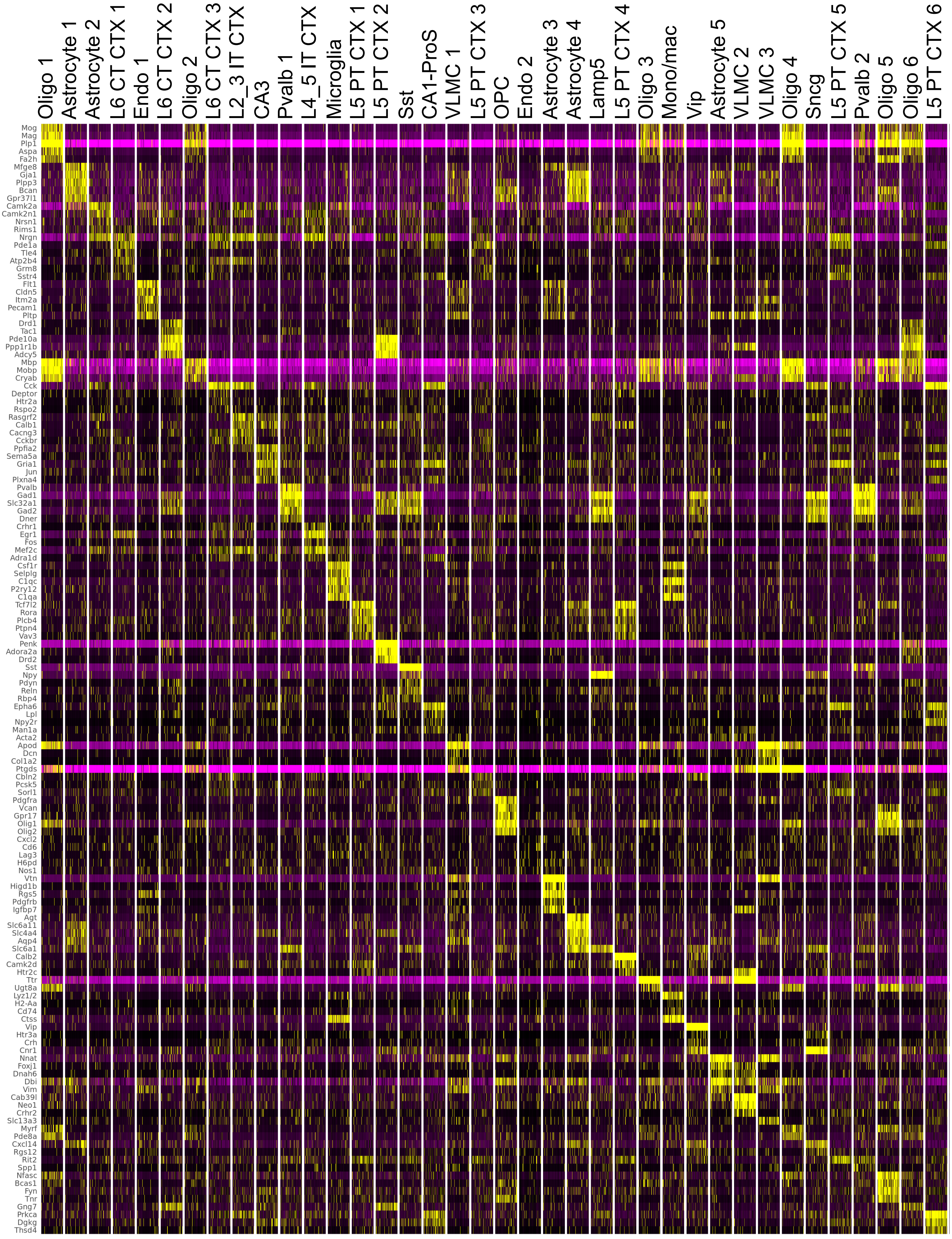

Supplemental Fig. 9: Heatmap of top 5 marker genes for all spatial transcriptomics subclusters, WT and all *Hexb*$^{-/-}$ groups.

(a) Heatmap of top 5 marker genes for each subcluster. DGE analysis was performed between each subcluster compared to all other subclusters to identify top 5 genes enriched in each subcluster.

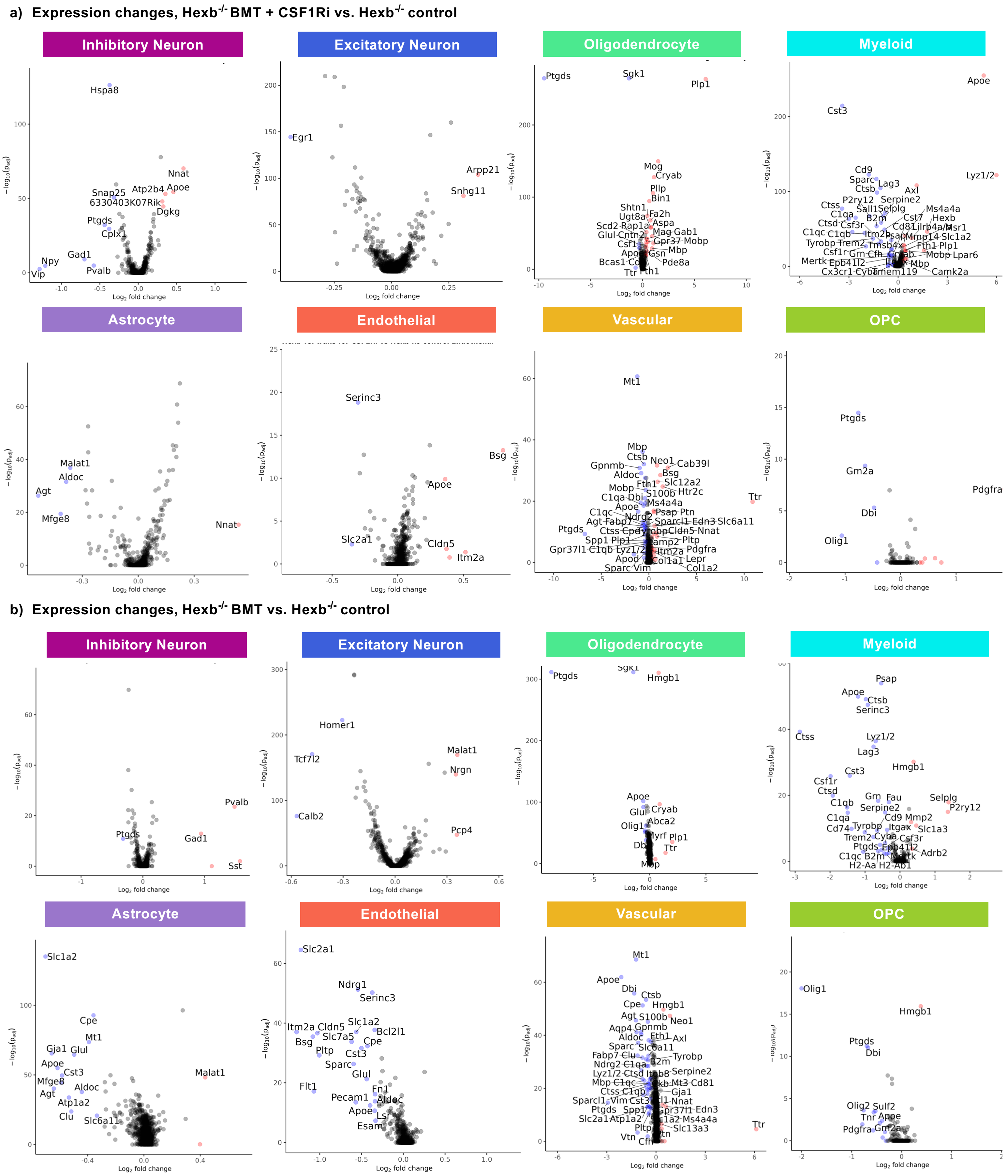
a) Expression changes, Hexb-/- BMT + CSF1Ri vs. Hexb-/- control
Inhibitory Neuron
Hspa8
Nnat
Snap25
Atp2b4
Apoe
6330403K07Rik
Dgkg
Ptgds
Cplx1
Gad1
Npy
Vip
Pvalb
Excitatory Neuron
Egr1
Arpp21
Snhg11
Oligodendrocyte
Ptgds
Sgk1
Plp1
Mog
Cryab
Pllp
Bin1
Shtn1
Ugt8a
Fa2h
Scd2
Rap1a
Aspa
Glul
Cntn2
Mag
Gab1
Csf1
Gpr37
Mobp
Gsn
Mbp
Bcas1
Pde8a
Ttr
Fth1
Myeloid
Apoe
Cst3
Lyz1/2
Cd9
Sparc
Lag3
Ctsb
Axl
P2ry12
Serpine2
Ctss
Sall1
Selplg
Ms4a4a
C1qa
B2m
Cst7
Hexb
Ctsd
Csf3r
Cd81
Lilrb4a/b
Msr1
C1qc
C1qb
Itm2b
Psap
Mmp14
Slc1a2
Tyrobp
Trem2
Tmsb4x
Fth1
Plp1
Csf1r
Grn
Cfh
Mobp
Lpar6
Mertk
Epb41l2
Mbp
Cx3cr1
Cyba
Tmem119
Camk2a
Astrocyte
Malat1
Aldoc
Agt
Mfge8
Nnat
Endothelial
Serinc3
Bsg
Apoe
Slc2a1
Cldn5
Itm2a
Vascular
Mt1
Mbp
Ctsb
Neo1
Cab39l
Gpnmb
Aldoc
Bsg
Fth1
Slc12a2
Mobp
S100b
Htr2c
C1qa
Dbi
Ms4a4a
Apoe
C1qc
Ndrg2
Psap
Ptn
Agt
Fabp7
Sparcl1
Edn3
Slc6a11
Ptgds
Ctss
Cldn5
Nnat
Ttr
Spp1
Plp1
Pltp
Gpr37l1
C1qb
Lyz1/2
Itm2a
Pdgfra
Apod
Col1a1
Lepr
Sparc
Vim
Col1a2
OPC
Ptgds
Gm2a
Pdgfra
Dbi
Olig1
Log2 fold change
− log10(padj)
b) Expression changes, Hexb-/- BMT vs. Hexb-/- control
Inhibitory Neuron
Pvalb
Ptgds
Gad1
Sst
Excitatory Neuron
Homer1
Tcf7l2
Malat1
Nrgn
Calb2
Pcp4
Oligodendrocyte
Ptgds
Sgk1
Hmgb1
Apoe
Cryab
Glul
Abca2
Olig1
Myrf
Plp1
Dbi
Ttr
Mbp
Myeloid
Psap
Apoe
Ctsb
Serinc3
Ctss
Lyz1/2
Lag3
Hmgb1
Cst3
Csf1r
Ctsd
Grn
Fau
Selplg
C1qb
Serpine2
P2ry12
C1qa
Cd9
Mmp2
Cd74
Tyrobp
Itgax
Slc1a3
Trem2
Cyba
Csf3r
Ptgds
Epb41l2
C1qc
B2m
Mertk
Adrb2
H2-Aa
H2-Ab1
Astrocyte
Slc1a2
Cpe
Mt1
Gja1
Glul
Apoe
Cst3
Malat1
Mfge8
Aldoc
Agt
Atp1a2
Clu
Slc6a11
Endothelial
Slc2a1
Ndrg1
Serinc3
Slc1a2
Itm2a
Cldn5
Bcl2l1
Slc7a5
Cpe
Bsg
Pltp
Cst3
Sparc
Glul
Flt1
Fn1
Pecam1
Aldoc
Apoe
Lsr
Esam
Vascular
Mt1
Apoe
Dbi
Ctsb
Cpe
Hmgb1
Agt
S100b
Neo1
Aqp4
Gpnmb
Aldoc
Fth1
Axl
Sparc
Slc6a11
Fabp7
Clu
Tyrobp
Ndrg2
C1qa
B2m
Lyz1/2
Ctsd
Serpine2
Mbp
C1qc
Mt3
Cd81
Ctss
C1qb
Gja1
Sparcl1
Vim
Cst3
Nnat
Ptgds
Spp1
Edn3
Slc2a1
Atp1a2
Ms4a4a
Slc13a3
Ttr
Pltp
Vtn
Cfh
OPC
Olig1
Hmgb1
Ptgds
Dbi
Olig2
Sulf2
Tnr
Apoe
Pdgfra
Gm2a
Log2 fold change
− log10(padj)

Supplemental Fig. 10: Spatial transcriptomics broad cell type differentially expressed genes, treated *Hexb*$^{-/-}$ versus *Hexb*$^{-/-}$ control. Volcano plots of DEGs between (a) BMT + CSF1Ri-treated *Hexb*$^{-/-}$ and *Hexb*$^{-/-}$ control and (b) BMT-treated *Hexb*$^{-/-}$ and *Hexb*$^{-/-}$ control for each broad cell type.

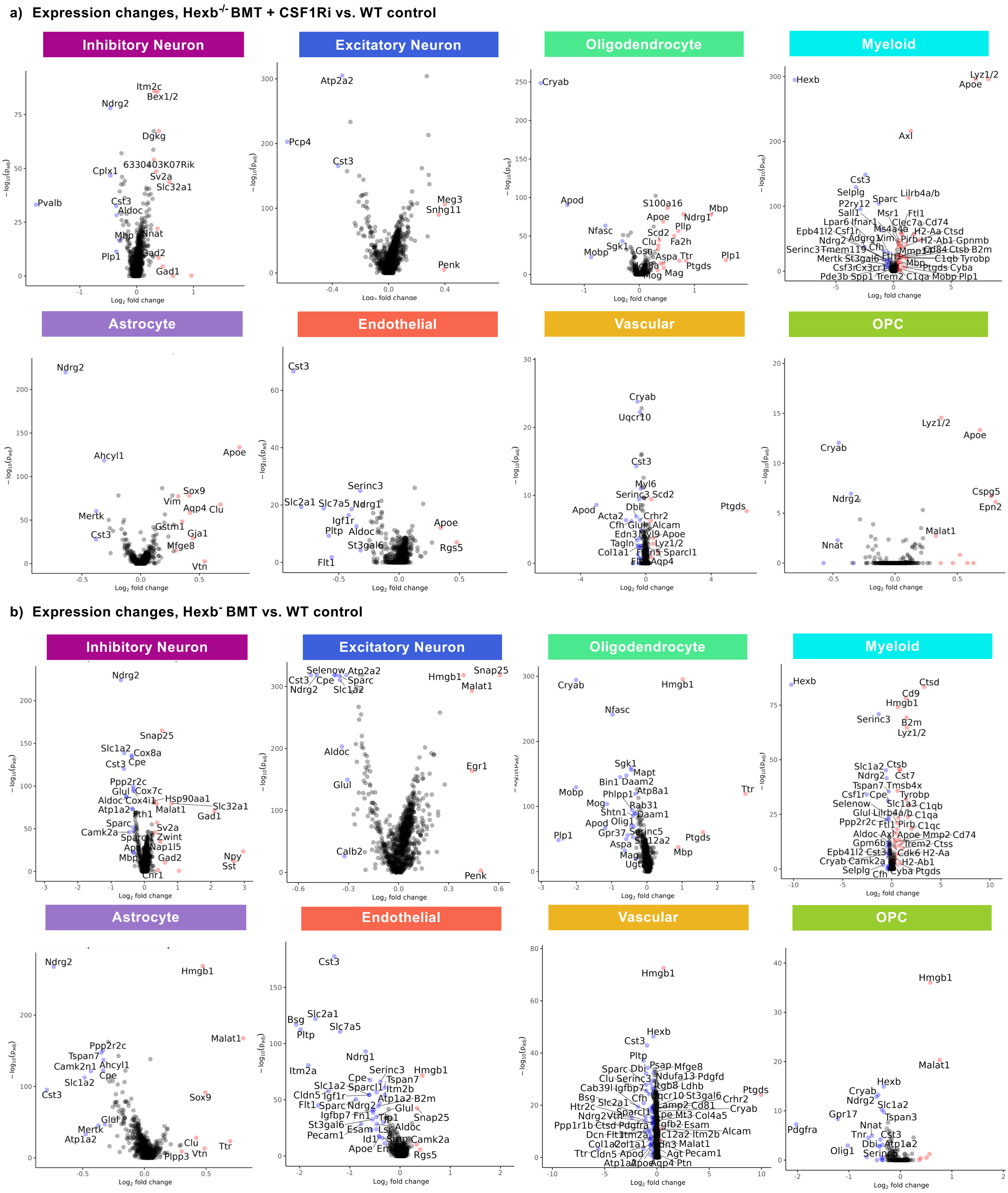
a) Expression changes, Hexb-/- BMT + CSF1Ri vs. WT control
Inhibitory Neuron
Excitatory Neuron
Oligodendrocyte
Myeloid
Astrocyte
Endothelial
Vascular
OPC
b) Expression changes, Hexb- BMT vs. WT control
Inhibitory Neuron
Excitatory Neuron
Oligodendrocyte
Myeloid
Astrocyte
Endothelial
Vascular
OPC
−log10(padj)
Log2 fold change
Itm2c
Bex1/2
Ndrg2
Dgkg
6330403K07Rik
Cplx1
Sv2a
Slc32a1
Pvalb
Cst3
Aldoc
Mbp
Nnat
Plp1
Gad2
Gad1
Atp2a2
Pcp4
Meg3
Snhg11
Penk
Cryab
Apod
S100a16
Mbp
Ndrg1
Nfasc
Apoe
Pllp
Scd2
Fa2h
Sgk1
Clu
Mobp
Gsn
Aspa
Ttr
Ptgds
Mag
Hexb
Lyz1/2
Apoe
Axl
Sparc
Lilrb4a/b
Selplg
Ahcyl1
Sox9
Vim
Aqp4
Clu
Mertk
Gstm1
Gja1
Mfge8
Vtn
Serinc3
Slc2a1
Slc7a5
Igf1r
Pltp
Aldoc
St3gal6
Rgs5
Flt1
Uqcr10
Myl6
Acta2
Aqp4
Cspg5
Epn2
Malat1
Nnat
Snap25
Hmgb1
Egr1
Calb2
Glul
Npy
Sst
Ctsd
Cd9
B2m
Pdgfra
Gpr17
Olig1

Supplemental Fig. 11: Spatial transcriptomics broad cell type differentially expressed genes, treated *Hexb*$^{-/-}$ versus WT^{-} control. Volcano plots of DEGs between (a) BMT + CSF1Ri-treated *Hexb*$^{-/-}$ and WT^{-} control and (b) BMT-treated *Hexb*$^{-/-}$ and WT control for each broad cell type.

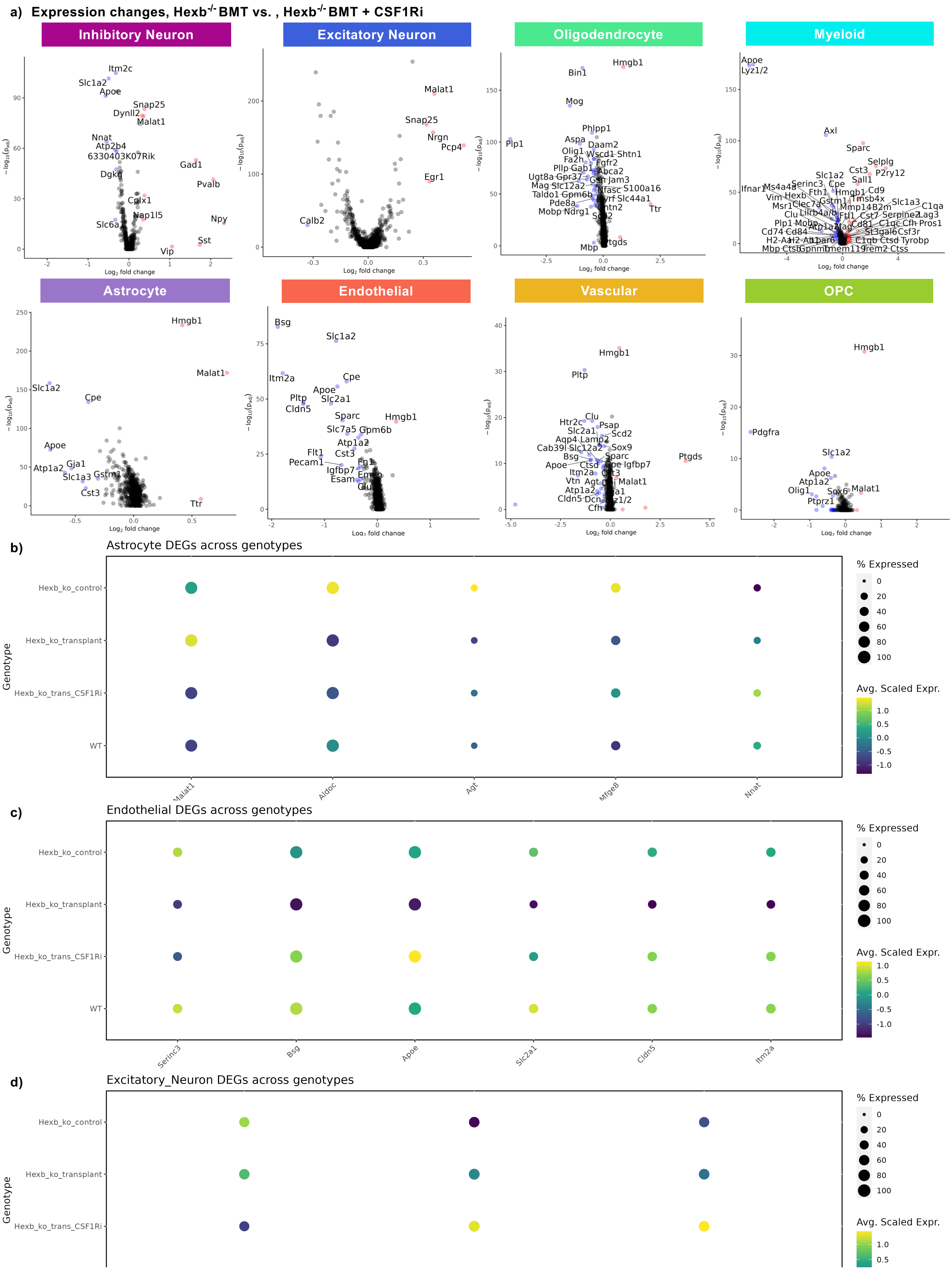
a) Expression changes, Hexb-/- BMT vs. , Hexb-/- BMT + CSF1Ri
Inhibitory Neuron
Excitatory Neuron
Oligodendrocyte
Myeloid
Astrocyte
Endothelial
Vascular
OPC
Itm2c
Slc1a2
Apoe
Snap25
Dynll2
Malat1
Nnat
Atp2b4
6330403K07Rik
Gad1
Dgkg
Pvalb
Cplx1
Nap1l5
Npy
Slc6a1
Sst
Vip
Nrgn
Pcp4
Egr1
Calb2
Hmgb1
Bin1
Mog
Plp1
Phlpp1
Aspa
Daam2
Olig1
Ttr
Mbp
Ptgds
Axl
Sparc
Selplg
P2ry12
Cst3
Sall1
Hexb
Cpe
Gja1
Slc1a3
Gstm1
Bsg
Itm2a
Pltp
Cldn5
Slc2a1
Slc7a5
Gpm6b
Atp1a2
Flt1
Pecam1
Igfbp7
Esam
Clu
Pdgfra
Olig1
Ptprz1
Sox6
Log₂ fold change
−log₁₀(p_adj)
b) Astrocyte DEGs across genotypes
Hexb_ko_control
Hexb_ko_transplant
Hexb_ko_trans_CSF1Ri
WT
Genotype
Malat1
Aldoc
Agt
Mfge8
Nnat
% Expressed
0
20
40
60
80
100
Avg. Scaled Expr.
1.0
0.5
0.0
-0.5
-1.0
c) Endothelial DEGs across genotypes
Hexb_ko_control
Hexb_ko_transplant
Hexb_ko_trans_CSF1Ri
WT
Genotype
Serinc3
Bsg
Apoe
Slc2a1
Cldn5
Itm2a
% Expressed
0
20
40
60
80
100
Avg. Scaled Expr.
1.0
0.5
0.0
-0.5
-1.0
d) Excitatory_Neuron DEGs across genotypes
Hexb_ko_control
Hexb_ko_transplant
Hexb_ko_trans_CSF1Ri
Genotype
% Expressed
0
20
40
60
80
100
Avg. Scaled Expr.
1.0
0.5

Supplemental Fig. 12: Spatial transcriptomics broad cell type differentially expressed genes, BMT-treated *Hexb*$^{-/-}$ versus BMT-treated *Hexb*$^{-/-}$, and broad cell type pseudobulk analysis, WT and all *Hexb*$^{-/-}$ groups. (a) Volcano plots of DEGs between BMT-treated *Hexb*$^{-/-}$ and BMT + CSF1Ri-treated *Hexb*$^{-/-}$ for each broad cell type. (b-d) Dot plots representing pseudo-bulked expression values across the four animal groups (WT control, *Hexb*$^{-/-}$ control, *Hexb*$^{-/-}$ BMT-treated, and *Hexb*$^{-/-}$ BMT + CSF1Ri-treated). Pseudo-bulk analysis was performed to identify the top DEGs in the (b) astrocyte, (c) endothelial cell, and (d) excitatory neuron broad cell types and plotted across the four animal groups.

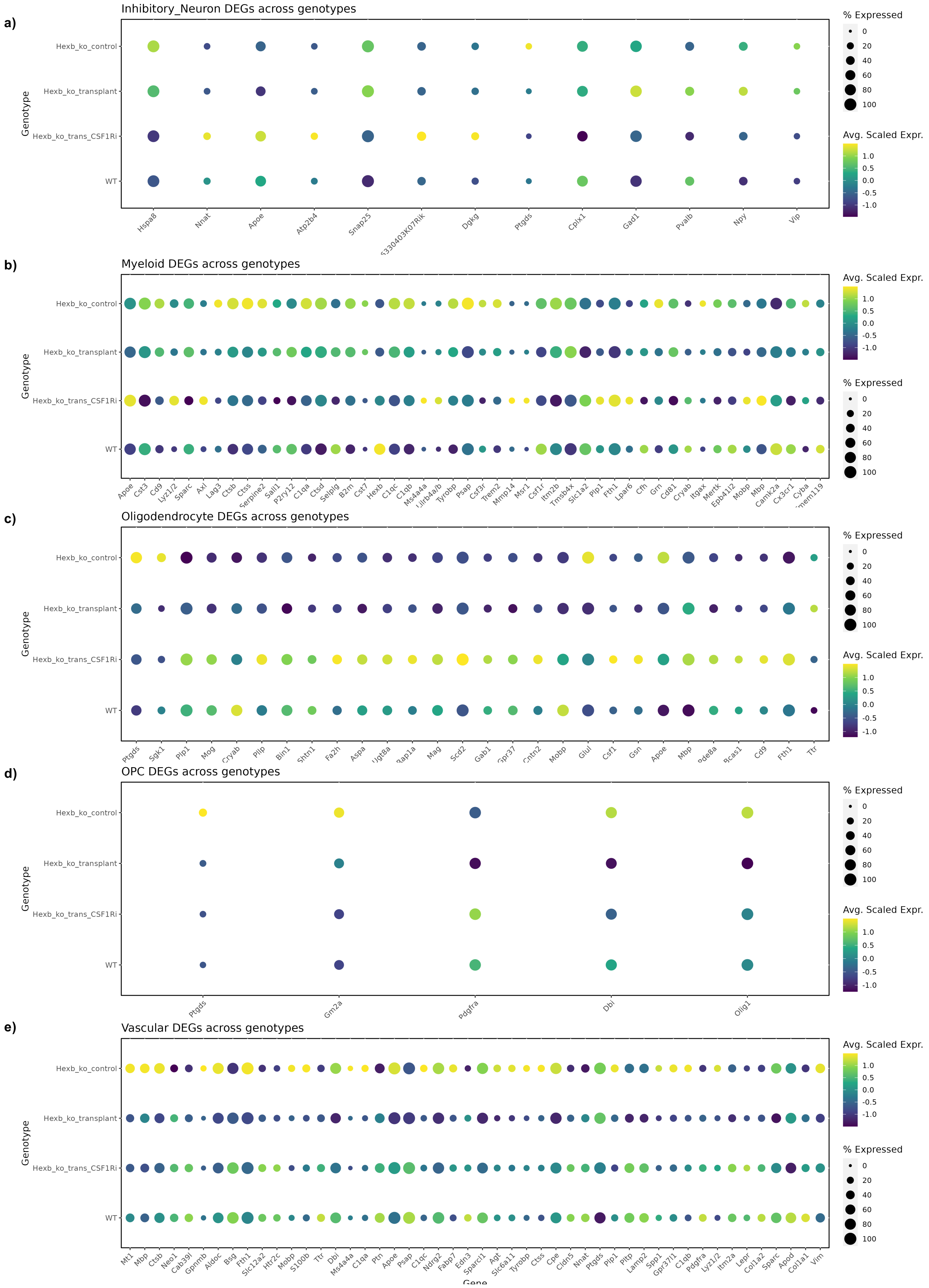

a)
Inhibitory_Neuron DEGs across genotypes
Genotype
Hexb_ko_control
Hexb_ko_transplant
Hexb_ko_trans_CSF1Ri
WT
Hspa8
Nnat
Apoe
Atp2b4
Snap25
Dgkg
Ptgds
Cplx1
Gad1
Pvalb
Npy
Vip
% Expressed
0
20
40
60
80
100
Avg. Scaled Expr.
1.0
0.5
0.0
-0.5
-1.0
b)
Myeloid DEGs across genotypes
Apoe
Cst3
Cd9
Lyz1/2
Sparc
Axl
Lag3
Ctsb
Ctss
Serpine2
Sall1
P2ry12
C1qa
Ctsd
Selplg
B2m
Cst7
Hexb
C1qc
C1qb
Ms4a4a
Tyrobp
Psap
Csf3r
Trem2
Mmp14
Msr1
Csf1r
Itm2b
Tmsb4x
Slc1a2
Plp1
Fth1
Lpar6
Cfh
Grn
Cd81
Cryab
Itgax
Mertk
Epb41l2
Mobp
Mbp
Camk2a
Cx3cr1
Cyba
c)
Oligodendrocyte DEGs across genotypes
Ptgds
Sgk1
Plp1
Mog
Cryab
Pllp
Bin1
Shtn1
Fa2h
Aspa
Mag
Scd2
Gab1
Mobp
Glul
Csf1
Gsn
Apoe
Mbp
Bcas1
Cd9
Fth1
Ttr
d)
OPC DEGs across genotypes
Ptgds
Gm2a
Pdgfra
Dbi
Olig1
e)
Vascular DEGs across genotypes
Mt1
Mbp
Ctsb
Neo1
Cab39l
Gpnmb
Aldoc
Bsg
Fth1
Slc12a2
Htr2c
Mobp
S100b
Ttr
Dbi
Ms4a4a
C1qa
Ptn
Apoe
Psap
C1qc
Ndrg2
Fabp7
Edn3
Sparcl1
Agt
Slc6a11
Tyrobp
Ctss
Cpe
Cldn5
Nnat
Ptgds
Plp1
Pltp
Lamp2
Spp1
Gpr37l1
C1qb
Pdgfra
Lyz1/2
Itm2a
Lepr
Col1a2
Sparc
Apod
Col1a1
Vim

Supplemental Fig. 13: Spatial transcriptomics broad cell type pseudobulk analysis, WT and all *Hexb*$^{-/-}$ groups. Dot plots representing pseudo-bulked expression values across the four animal groups (WT control, *Hexb*$^{-/-}$ control, BMT-treated *Hexb*$^{-/-}$, and BMT + CSF1Ri-treated *Hexb*$^{-/-}$). Pseudo-bulk analysis was performed to identify the top DEGs in the (a) inhibitory neuron, (b) myeloid, (c) oligodendrocyte, (d) oligodendrocyte precursor cell, and (e) vascular broad cell types and plotted across the four animal groups.

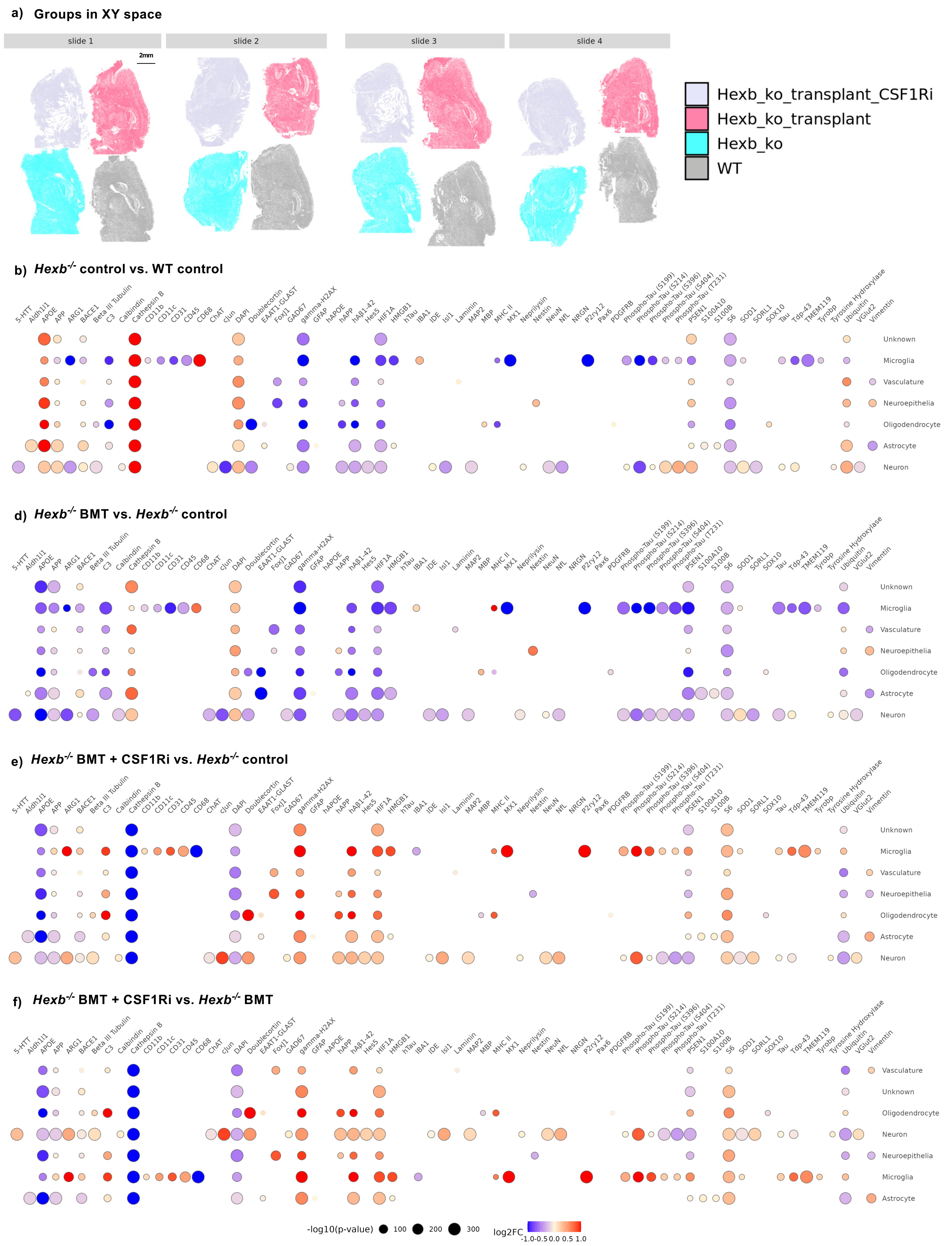
a) Groups in XY space
slide 1
slide 2
slide 3
slide 4
2mm
Hexb_ko_transplant_CSF1Ri
Hexb_ko_transplant
Hexb_ko
WT
b) Hexb-/- control vs. WT control
d) Hexb-/- BMT vs. Hexb-/- control
e) Hexb-/- BMT + CSF1Ri vs. Hexb-/- control
f) Hexb-/- BMT + CSF1Ri vs. Hexb-/- BMT
5-HTT
Aldh1l1
APOE
APP
ARG1
BACE1
Beta III Tubulin
C3
Calbindin
Cathepsin B
CD11b
CD11c
CD31
CD45
CD68
ChAT
cJun
DAPI
Doublecortin
EAAT1-GLAST
FoxJ1
GAD67
gamma-H2AX
GFAP
hAPOE
hAPP
hAβ1-42
Hes5
HIF1A
HMGB1
hTau
IBA1
IDE
Isl1
Laminin
MAP2
MBP
MHC II
MX1
Neprilysin
Nestin
NeuN
NfL
NRGN
P2ry12
Pax6
PDGFRB
Phospho-Tau (S199)
Phospho-Tau (S214)
Phospho-Tau (S396)
Phospho-Tau (S404)
Phospho-Tau (T231)
PSEN1
S100A10
S100B
S6
SOD1
SORL1
SOX10
Tau
Tdp-43
TMEM119
Tyrobp
Tyrosine Hydroxylase
Ubiquitin
VGlut2
Vimentin
Unknown
Microglia
Vasculature
Neuroepithelia
Oligodendrocyte
Astrocyte
Neuron
-log10(p-value)
100
200
300
log2FC
-1.0 -0.5 0.0 0.5 1.0

Supplemental Fig. 14: Expanded spatial proteomics data visualization. (a) Projection of all brains (WT control, *Hexb*$^{-/-}$ control, BMT-treated *Hexb*$^{-/-}$, and BMT + CSF1Ri-treated *Hexb*$^{-/-}$) in XY space. 4 brains per group (n=16) across 4 slides with 4 brains per slide. (b-f) Bubble plots of differentially expressed proteins (DEPs) of interest in broad cell types between pairs (b) *Hexb*$^{-/-}$ control vs. WT control, (c) BMT-treated *Hexb*$^{-/-}$ vs. *Hexb*$^{-/-}$ control, (d) BMT + CSF1Ri-treated *Hexb*$^{-/-}$ vs. *Hexb*$^{-/-}$ control, and (e) BMT + CSF1Ri-treated *Hexb*$^{-/-}$ vs. BMT-treated *Hexb*$^{-/-}$. Dots are sized by p value ($-\log_{10}$p value) and colored by average difference ($\log_2$ fold change, red indicating increased expression, blue indicating decreased expression) of each DEP.

Representative images of inhibition of extracellular Hexβ uptake into neuronal lysosomes *in vitro*

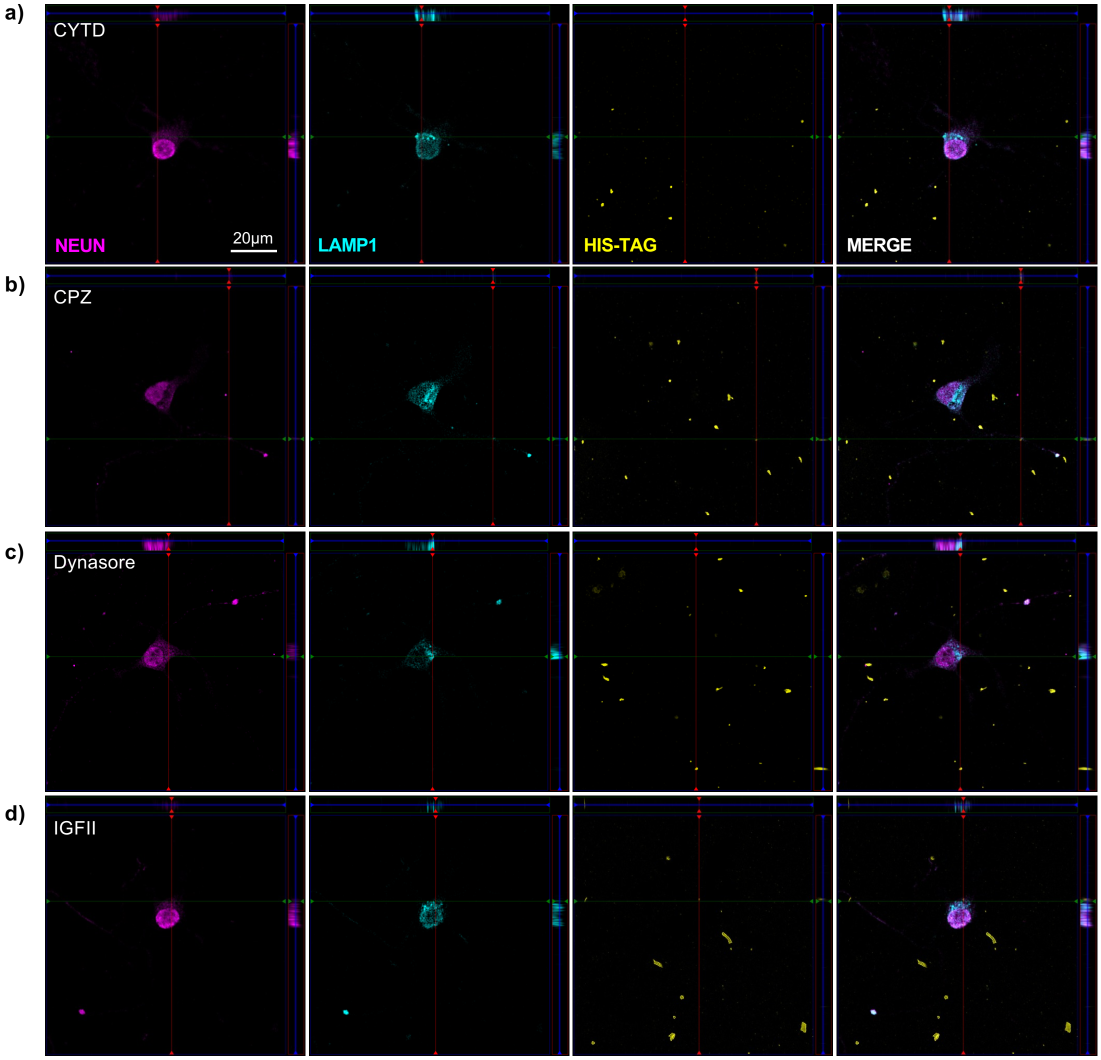

Supplemental Fig. 15: Representative images demonstrating inhibition of extracellular Hexβ uptake into neuronal lysosomes *in vitro*. Confocal images of mouse hippocampal neurons pretreated with endocytosis inhibitors (a) 100nM Cytochalasin D (CYTD), (b) 10μM Chlorpromazine (CPZ), (c) 80μM dynasore, and (d) 25ng/ml IGFII for 1h, followed by incubation with 10μg of his-tagged recombinant Hexβ protein for 24h. Cultures were immunolabeled for neurons (NeuN, magenta), lysosome-associated membrane protein 1 (LAMP1, cyan), his-tagged Hexβ protein (HIS-TAG, yellow), and a merged image showing orthogonal x/z and z/y projections at top and right of image showing colocalization of LAMP1$^+$ and HIS-TAG$^+$ staining within NeuN$^+$ neurons (white).
