# Supplementary material for: Microglial replacement in a Sandhoff disease mouse model reveals myeloid-derived β-hexosaminidase is necessary for neuronal health
Source: Nat Commun. 2025 Aug 27;16:7994. doi: 10.1038/s41467-025-63237-0 (PMC12391554; doi:10.1038/s41467-025-63237-0)
Supplement: Supplementary file 2 — Reporting Summary [file 41467_2025_63237_MOESM2_ESM.pdf]

nature portfolio

Corresponding author(s): Dr. Kim N. Green
Last updated by author(s): Jul 31, 2025

Reporting Summary

Nature Portfolio wishes to improve the reproducibility of the work that we publish. This form provides structure for consistency and transparency in reporting. For further information on Nature Portfolio policies, see our Editorial Policies and the Editorial Policy Checklist.

Statistics

For all statistical analyses, confirm that the following items are present in the figure legend, table legend, main text, or Methods section.

n/a	Confirmed	
☐	☒	The exact sample size (*n*) for each experimental group/condition, given as a discrete number and unit of measurement
☐	☒	A statement on whether measurements were taken from distinct samples or whether the same sample was measured repeatedly
☐	☒	The statistical test(s) used AND whether they are one- or two-sided<br>*Only common tests should be described solely by name; describe more complex techniques in the Methods section.*
☐	☒	A description of all covariates tested
☐	☒	A description of any assumptions or corrections, such as tests of normality and adjustment for multiple comparisons
☐	☒	A full description of the statistical parameters including central tendency (e.g. means) or other basic estimates (e.g. regression coefficient) AND variation (e.g. standard deviation) or associated estimates of uncertainty (e.g. confidence intervals)
☐	☒	For null hypothesis testing, the test statistic (e.g. *F*, *t*, *r*) with confidence intervals, effect sizes, degrees of freedom and *P* value noted<br>*Give P values as exact values whenever suitable.*
☒	☐	For Bayesian analysis, information on the choice of priors and Markov chain Monte Carlo settings
☒	☐	For hierarchical and complex designs, identification of the appropriate level for tests and full reporting of outcomes
☒	☐	Estimates of effect sizes (e.g. Cohen's *d*, Pearson's *r*), indicating how they were calculated

Our web collection on statistics for biologists contains articles on many of the points above.

Software and code

Policy information about availability of computer code

Data collection	Commercial software: Nanostring AtomX Software, RNA Quality Control Module
Data analysis	Open-source R packages: R 4.3.1 software, Seurat 5.0.1 SCTransform, ggplot2 3.4.4, MAST (Model-based Analysis of Single-cell Transcriptomes)

For manuscripts utilizing custom algorithms or software that are central to the research but not yet described in published literature, software must be made available to editors and reviewers. We strongly encourage code deposition in a community repository (e.g. GitHub). See the Nature Portfolio guidelines for submitting code & software for further information.

Data

Policy information about availability of data

All manuscripts must include a data availability statement. This statement should provide the following information, where applicable:

- Accession codes, unique identifiers, or web links for publicly available datasets
- A description of any restrictions on data availability
- For clinical datasets or third party data, please ensure that the statement adheres to our policy

Gene and protein expression data produced by CosMx spatial molecular imaging are accessible as .rds files at https://doi.org/10.5061/dryad.3tx95x6rq

Research involving human participants, their data, or biological material

Policy information about studies with human participants or human data. See also policy information about sex, gender (identity/presentation), and sexual orientation and race, ethnicity and racism.

Reporting on sex and gender	*Use the terms sex (biological attribute) and gender (shaped by social and cultural circumstances) carefully in order to avoid confusing both terms. Indicate if findings apply to only one sex or gender; describe whether sex and gender were considered in study design; whether sex and/or gender was determined based on self-reporting or assigned and methods used. Provide in the source data disaggregated sex and gender data, where this information has been collected, and if consent has been obtained for sharing of individual-level data; provide overall numbers in this Reporting Summary. Please state if this information has not been collected. Report sex- and gender-based analyses where performed, justify reasons for lack of sex- and gender-based analysis.*
Reporting on race, ethnicity, or other socially relevant groupings	*Please specify the socially constructed or socially relevant categorization variable(s) used in your manuscript and explain why they were used. Please note that such variables should not be used as proxies for other socially constructed/relevant variables (for example, race or ethnicity should not be used as a proxy for socioeconomic status). Provide clear definitions of the relevant terms used, how they were provided (by the participants/respondents, the researchers, or third parties), and the method(s) used to classify people into the different categories (e.g. self-report, census or administrative data, social media data, etc.) Please provide details about how you controlled for confounding variables in your analyses.*
Population characteristics	*Describe the covariate-relevant population characteristics of the human research participants (e.g. age, genotypic information, past and current diagnosis and treatment categories). If you filled out the behavioural & social sciences study design questions and have nothing to add here, write "See above."*
Recruitment	*Describe how participants were recruited. Outline any potential self-selection bias or other biases that may be present and how these are likely to impact results.*
Ethics oversight	*Identify the organization(s) that approved the study protocol.*

Note that full information on the approval of the study protocol must also be provided in the manuscript.

Field-specific reporting

Please select the one below that is the best fit for your research. If you are not sure, read the appropriate sections before making your selection.

☒ Life sciences ☐ Behavioural & social sciences ☐ Ecological, evolutionary & environmental sciences

For a reference copy of the document with all sections, see nature.com/documents/nr-reporting-summary-flat.pdf

Life sciences study design

All studies must disclose on these points even when the disclosure is negative.

Sample size	An n of at least 10 per group for all bone marrow transplant experiments was selected considering commonly utilized group sizes within the literature, while maintaining an equal sex distribution of an n of 5 per sex per group in order to fully explore sex as a biological variable.
Data exclusions	Significance of single outliers was calculated using the Grubbs' extreme studentized deviate method and significant ($p < 0.05$) outliers were excluded.
Replication	Mice were transplanted in 11 distinct replicate cohorts to assess reproducibility of treatment. Cell culture experiments were repeated 3-4 times each to ensure consistency of results. All attempts at replication were successful and produced consistent results.
Randomization	We employed a randomization strategy such that mice of appropriate genotype were randomly placed in the control and experimental group (s).
Blinding	Experimenters were blinded to genotype and treatment condition for all behavioral assays and analysis of histological images.

Reporting for specific materials, systems and methods

We require information from authors about some types of materials, experimental systems and methods used in many studies. Here, indicate whether each material, system or method listed is relevant to your study. If you are not sure if a list item applies to your research, read the appropriate section before selecting a response.

Materials & experimental systems

n/a	Involved in the study
☐	☒ Antibodies
☒	☐ Eukaryotic cell lines
☒	☐ Palaeontology and archaeology
☐	☒ Animals and other organisms
☒	☐ Clinical data
☒	☐ Dual use research of concern
☒	☐ Plants

Methods

n/a	Involved in the study
☒	☐ ChIP-seq
☐	☒ Flow cytometry
☒	☐ MRI-based neuroimaging

Antibodies

Antibodies used

Flow Cytometry: Cells were stained for flow cytometric analysis with the following surface antibodies purchased from Biolegend (San Diego, CA) and diluted in PBS at 1:200 unless otherwise noted: CD34 (eFlour660 (1:50, #50-0341-80, eBioscience), PE (Invitrogen, 1:100, #PIMA517831)), Sca-1-AF700 (1:100, #108141), Ter119-PE/Cy5 (#116209), ckit/CD117-PE/Cy7 (#25-1171-81, eBioscience), CD150/SLAM (PerCP-eFlour710 (#46-1502-82, eBioscience), BV605 (BD Horizon, 1:100, #BDB567309)), CD11b-APC (#101212), Gr1/Ly6C-AF700 (#108422), CD45-APC/Cy7 (1:100, #103116), NK1.1-PE (#108707), CD27-APC/Cy7 (#124226), DAPI (1:400, #422801), CD3-BV421 (1:100, #100227), CD19-BV421 (1:100, #115537), Ly6G-PE/Cy7 (1:100, #127617), CCR2-PE (1:100, #150609), CD16/32-BV605 (BD Horizon, 1:400, #BDB563006).

Immunohistochemistry: Brain and liver sections were stained with combinations of antibodies against ionized calcium-binding adapter molecule 1 (IBA1, 1:1000; #019–19741, Wako, Osaka, Japan), glial fibrillary acidic protein (GFAP; 1:1000; AB134436; Abcam, Cambridge, MA, United States, green fluorescent protein (GFP, 1:200; ab13970, Abcam, Waltham, MA), neuronal nuclei (NeuN, 1:1000; Ab104225; Abcam), lysosome-associated membrane protein 1 (LAMP1, 1:200; Ab25245, Abcam), and parvalbumin (Pvalb, 1:500; MAB1572, Millipore, Burlington, MA). Sections were then stained with secondary antibodies secondary antibodies Alexa Fluor 633 (A21094, Thermofisher), Alexa Fluor 555 (A21422, Thermofisher) and Alexa Fluor 488 (A11034, Thermofisher), at a 1:200 concentration.

Cell culture immunohistochemistry: Cells were stained with a combination of rat Anti- mouse LAMP1 (1:250; Ab25245, ABCAM), Rabbit anti- 6x his-tag (1:500; MA5-33032, Invitrogen) and mouse anti- mouse NeuN (1:500; MA5, 33103, Invitrogen) primary antibodies and secondary antibodies Alexa Fluor 633 (A21094, Thermofisher), Alexa Fluor 555 (A21422, Thermofisher) and Alexa Fluor 488 (A11034, Thermofisher), at a 1:250 concentration.

Validation

All antibodies were validated by the manufacturers for use on mouse-derived samples for their respective applications.

Animals and other research organisms

Policy information about studies involving animals; ARRIVE guidelines recommended for reporting animal research, and Sex and Gender in Research

Laboratory animals

All mice were obtained from The Jackson Laboratory. We utilized B6;129S-Hexbtm1Rlp/J mice in this study, which harbor a loss-of-function mutation in the Hexb gene strain #002914. Mice were sacrificed at 16 weeks of age. Bone marrow cells were isolated from sex-matched CAG-EGFP donor mice less than or equal to 5 months of age (strain #006567).

Wild animals

n/a

Reporting on sex

Sex was fully considered as a biological variable with an n of 5 per sex per group. Statistical analysis was first performed with sexes separated to assess sex differences. No significant sex differences were detected on any behavioral assay or pathological readout as assessed with histology.

Field-collected samples

n/a

Ethics oversight

Institutional Animal Care and Use Committee (IACUC) at the University of California, Irvine

Note that full information on the approval of the study protocol must also be provided in the manuscript.

Plants

Seed stocks	n/a
Novel plant genotypes	n/a
Authentication	n/a

Flow Cytometry

Plots

Confirm that:

- [x] The axis labels state the marker and fluorochrome used (e.g. CD4-FITC).
- [x] The axis scales are clearly visible. Include numbers along axes only for bottom left plot of group (a 'group' is an analysis of identical markers).
- [x] All plots are contour plots with outliers or pseudocolor plots.
- [x] A numerical value for number of cells or percentage (with statistics) is provided.

Methodology

Sample preparation	At the time of sacrifice, bone marrow, whole blood, and/or whole brains were harvested and analyzed by flow cytometry for hematopoietic stem cell and granulocyte chimerism and/or brain parenchymal engrafted cell profiling. Bone marrow/ hematopoietic stem cells were extracted from femurs and tibia by flushing with ice cold PBS. Whole blood/granulocytes were collected in EDTA via cardiac puncture following CO2 euthanasia. Samples were centrifuged at 1250rpm for 5 minutes. Supernatant was discarded, then samples were incubated with 1 mL of 1x ACK Lysing Buffer (A1049201, Gibco, Waltham, MA) for 1 minute at RT, protected from light. Reaction was quenched with 9 mL of ice cold PBS, and cells were again centrifuged at 1250rpm for 5 minutes. Supernatant was discarded, and pellet was resuspended in 1mL PBS. Finally, samples were centrifuged at 2400rpm for 5 minutes, supernatant was discarded, and pellet was reconstituted in 225µL of PBS. Brains were dissociated using the Miltenyi Biotec Multi Tissue Dissociation Kit 1 (# 130-110-201, Milenyi, Auburn, CA) on a gentleMACS Octo Dissociator with Heaters (# 130-096-427, Miltenyi) following the Miltenyi protocol entitled "Dissociation of inflamed neural tissue using the Multi Tissue Dissociation Kit 1" to preserve immune cell epitopes for flow cytometric processing. Myelin and cell debris were removed using Debris Removal Solution (# 130-109-398, Miltenyi) without performing red blood cell lysis to preserve cell viability.
Instrument	Flow cytometry analysis was performed using a BD LSRFortessa X20 Benchtop Flow Cytometer (BD Biosciences, Franklin Lakes, NJ).
Software	Data was analyzed in BD FACSDiva, FlowJo, and FCS Express software.
Cell population abundance	At least 1,000,000 events were assessed for hematopoietic stem cells, 5,000,000 for granulocytes, and 5-10,000,000 for brain hemispheres. Propidium iodide or DAPI were used to assess the percentage of live cells.
Gating strategy	For chimerism assessment: cells were first gated to eliminate debris based on SSC and FSC; cells with low values were excluded. Doublets were gated out based on FSC-height and FSC-area. Cells were then gated as PI+ or PI- such that only live cells were considered. Granulocytes were defined as CD45+NK1.1−CD11b+GR1+ cells. Hematopoietic stem cells were defined as Ter119−CD27+ckit+Sca+CD150+CD34− cells. For engrafted myeloid cell identification, cells were gated on forward scatter area (FSC-A) and side scatter area (SSC-A) to remove debris and dead cells, forward scatter height (FSC-H) to remove doublets, DAPI/CD3/CD19 to remove dead cells, T cells, and B cells, GFP to remove non-donor-derived cells, CD45 to remove non-bone marrow-derived cells, and Cd11b to separate myeloid and non-myeloid cells. Cells were then gated on GFP to identify donor-derived cells. For panel #1 (progenitor panel), all GFP+CD45hi cells in both Cd11b+ and Cd11b- populations were gated on cKit and Sca-1. Cd11b-Sca1-cKit- cells were then gated on CD34. For panel #2, all GFP+CD45hiCd11b+ cells were gated on Ly6C and Ly6G. Ly6C-Ly6G-, Ly6C+Ly6G-, and Ly6C+Ly6G+ cells were then gated on CCR2 and CD16/32.

- [x] Tick this box to confirm that a figure exemplifying the gating strategy is provided in the Supplementary Information.
